# Visualizing Thiol Stress Responses in Cells with a Water-Soluble Raman Sensor

**DOI:** 10.1021/acs.analchem.5c04391

**Published:** 2025-11-10

**Authors:** Hiroyuki Yamakoshi, Meichen Wang, Keisuke Koga, Shinji Kajimoto, Yuse Kuriyama, Yusuke Sasano, Takaaki Akaike, Yoshiharu Iwabuchi, Takakazu Nakabayashi

**Affiliations:** † Graduate School of Pharmaceutical Sciences, 13101 Tohoku University, 6-3 Aoba, Aramaki, Aoba-ku, Sendai 980-8578, Japan; ‡ Department of Environmental Medicine and Molecular Toxicology, Graduate School of Medicine, Tohoku University, 2-1 Seiryo-machi, Aoba-ku, Sendai 980-8575, Japan

## Abstract

Accurate quantification of intracellular thiols in live cells remains challenging owing to the inherent properties of fluorescent probes, such as signal saturation, photobleaching, and dye aggregation, despite the development of numerous thiol-detecting probes. Here, we present (*E*)-2-cyano-3-isopropylacrylamide (iPrCAA) as a ratiometric Raman probe for quantifying and visualizing intracellular thiol concentrations. iPrCAA is the smallest thiol-detecting probe currently available for live-cell detection (molecular weight 138 g/mol). It exhibits high reactivity toward thiol-adduct formation and solubility in water, which enhance its sensitivity in relation to that of existing Raman probes. Thus, iPrCAA enables the real-time monitoring of thiol depletion in live cells, demonstrating that a thiol-free medium reduces thiol levels by approximately 3-fold. Through its ability to visualize endogenous molecules, iPrCAA revealed that the thiol distribution closely resembles that of proteins. This Raman probe provides a practical means for efficiently quantifying endogenous thiols and dynamically tracking thiol fluctuations under stress conditions.

## Graphical abstract

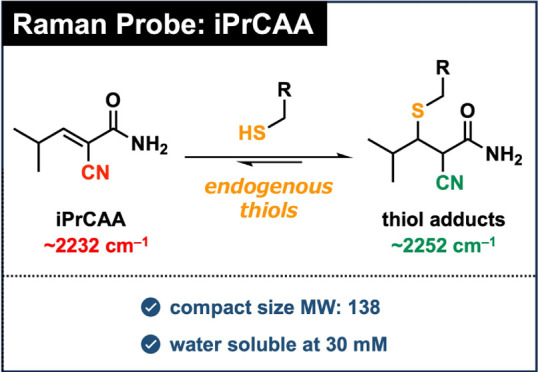

## Introduction

Live-cell imaging is a critical approach to elucidating biological processes. Although fluorescent probes are commonly used for such imaging, Raman probes have recently garnered interest.^1–4^ Unlike fluorescent probes, Raman probes use small functional groups such as alkynes or nitriles as markers.^4–6^ Their narrower bandwidth results in the achievement of higher resolution and more detailed structural information. However, because Raman signals are considerably weaker than fluorescence, the development of practical Raman probes remains a significant challenge.

The detection of intracellular ions and molecules using Raman probes is based on the peak shifts or intensity changes that result from interactions or chemical reactions. For example, in 2014, our group demonstrated that carbonyl cyanide *p*-trifluoromethoxyphenylhydrazone (FCCP), a weakly acidic nitrile compound, exhibits a Raman peak at 2226 cm^–1^ in the molecular form, whereas peaks at 2176 and 2195 cm^–1^ are observed in the ionic form. The two forms can thus be effectively distinguished for successful imaging.^7^ Wilson et al. expanded this concept to diyne compounds, thereby developing pH sensors.^8,9^ Zeng et al. used azides as the reactive functional group to detect hydrogen sulfide.^10^ Fujioka et al. developed an activatable Raman probe for visualizing multiple enzyme activities, leveraging the increase in Raman scattering intensity owing to the preresonance effect.^11,12^

In 2023, we developed ThioRas, a thiol-detecting probe based on a reversible thia-Michael reaction (Figure 1a).^13^ The conjugated nitrile group in ThioRas exhibits a Raman peak at 2237 cm^–1^, whereas the alkyl nitrile in the thiol adduct (TA) displays a shifted peak at 2259 cm^–1^. Quantifying the ratio of these two peaks enables the estimation of the thiol concentration. The low molecular weight of ThioRas (i.e., 167 g/mol) enables efficient diffusion and facilitates the simultaneous detection of externally introduced glutathione across various cellular compartments. However, their limited sensitivity prevents the detection of endogenous TAs.

**Figure 1:**
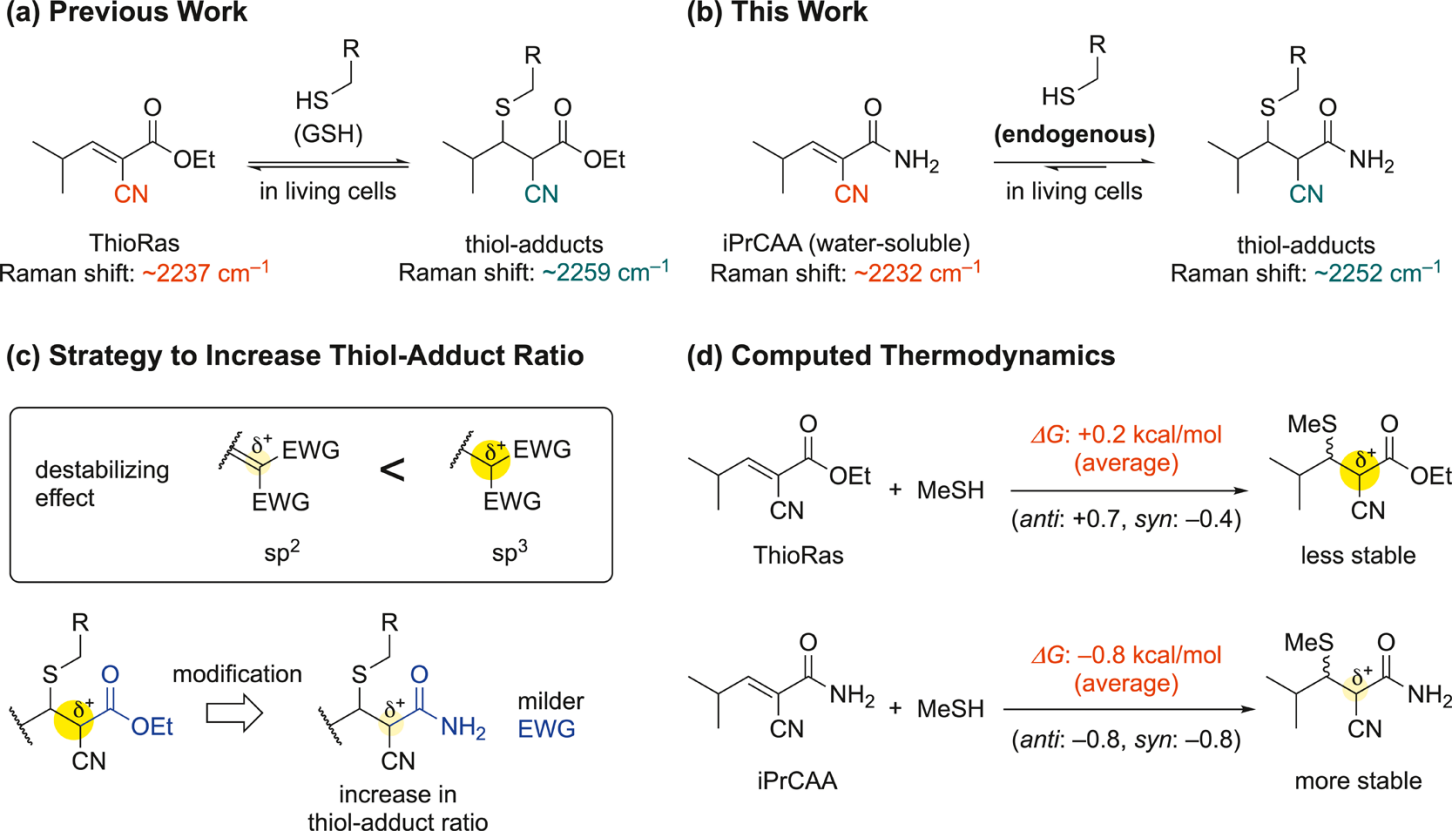
α-Cyanoacrylate-based Raman probes for ratiometric detection of thiols in live cells. (a) Reversible thia-Michael reaction of our previously reported probe, ThioRas. (b) Reversible thia-Michael reaction of our newly developed probe, iPrCAA. (c) Design of amide-based probes. (d) Computational prediction of thermodynamic properties in the reversible thia-Michael reaction. Δ*G* was computed at the M06-2X/6-311+G­(d,p) level of theory. (*E*)-2-cyano-3-isopropylacrylamide, iPrCAA.

To address this limitation, this study explores the use of 2-cyanoacrylamide (CAA), specifically the isopropyl analog (*E*)-2-cyano-3-isopropylacrylamide (iPrCAA),^14^ where the ethyl ester in ThioRas is replaced with a primary amide (Figure 1b). The Raman shifts of CAA and its TAs are similar to those of ThioRas and its TAs, suggesting that CAA exhibits similar thiol-detection capabilities. This modification, resulting in a smaller, less electron-withdrawing, and more polarized structure, is expected to increase the proportion of TAs and improve the water solubility. The combination of iPrCAA-based quantification with water as a reference offers a straightforward and reliable method for measuring endogenous thiol concentrations, providing a practical and accessible tool for live-cell analysis. This approach enables the precise and real-time monitoring of thiol fluctuations in live cells in response to various stimuli, which is crucial for studying biological processes. By leveraging this Raman probe, researchers can efficiently quantify endogenous thiols and monitor dynamic changes in thiol levels under stress conditions, allowing researchers to gain deeper insights into dynamic biochemical processes and paving the way for advancements in various fields.

## Materials and Methods

### Raman Microscopy

Raman spectra and images were obtained by using a confocal Raman system (Nanofinder flex2, Tokyo Instruments) equipped with an inverted microscope (Eclipse Ti2, Nikon). Details of the experimental setup for Raman microscopy can be found elsewhere.^15,16^ Briefly, the excitation beam from a 532 nm continuous-wave laser (Sprout Solo, Lighthouse Photonics) was focused onto a sample using a water-immersion objective lens (60×, 1.27 NA), and the Raman signal was collected through the same objective. The laser irradiation intensity and exposure time per point were 50 mW and 0.2 s, respectively, unless otherwise stated. The culture medium was replaced with Hank’s balanced salt solution (HBSS; H8264, Sigma) immediately before the measurement, and all measurements were conducted at 20 °C.

The Raman hyperspectral data set was processed using the singular-value decomposition (SVD) technique for noise reduction.^17^ Given the distinct autofluorescence-background signals at different points in the Raman spectrum, a modified polynomial-fitting technique^18^ was used to determine the baseline signal of autofluorescence, which was subtracted from the original Raman spectrum. Lastly, a Raman image was constructed on the basis of the intensity of each vibrational band of interest at each spatial position.

### Reversible Thia-Michael Reactions Monitored by Raman Microscopy

CAA (120 mM in DMSO) and 2-mercaptoethanol (400 mM in PBS, pH 7.4) were mixed in a 3:1 ratio. After 5 min, Raman spectra of the reaction mixture were recorded. The CAA:TA molar ratio was calculated from the relative intensity of each peak, corrected for intensity by the relative Raman intensity vs 5-ethynyl-2′-deoxyuridine (EdU) (RIE) values shown in Figure S1. As the TA could not be isolated, its RIE was approximated as 0.081, equivalent to that of alkyl nitrile **1**. The Raman shifts of the peaks are illustrated in the Supporting Information.

### Quantitative Analysis of Endogenous Thiols

An iPrCAA solution was freshly prepared in water at five times the final concentration and diluted 5-fold with HBSS to obtain the final concentration. The culture medium was washed twice with HBSS and then replaced with the prepared iPrCAA solution immediately before measurements. Raman imaging was performed as described in the “Raman microscopy” section.

The Raman spectra of the homogeneous solutions of iPrCAA at various concentrations were measured. The peak intensity of nitrile, normalized to the OH stretching band of water, was plotted against the iPrCAA concentration to obtain the calibration curve. To construct a calibration curve for thiol concentration, iPrCAA (final concentrations: 5, 10, 15, and 20 mM) and 2-mercaptoethanol (final concentrations: 1, 2, 4, 8, and 16 mM) were mixed in water. After 5 min, the Raman spectra were measured. The concentrations of iPrCAA and TA, as well as their ratios, were determined from the corresponding nitrile peak intensities. Thiol concentrations were determined by fitting the closest probe concentration and iPrCAA ratio to the calibration curve.

We applied hierarchical clustering analysis to the Raman images. Each cluster was assigned to medium, cytoplasm, nucleoplasm, nucleolus, or lipid droplet by comparing its spatial distribution with the corresponding bright-field image and analyzing the mean spectrum, particularly the intensities of CH and nucleic-acid Raman bands and resonance Raman bands of cytochrome *c*.^16,19^ SVD and fitting analyses were performed by using Igor Pro 8 (WaveMetrics).

## Results and Discussion

### Enhancement of the TA Ratio

Figure 1c illustrates a strategy to increase the TA ratio. Krenske et al. demonstrated that two electron-withdrawing groups on the same carbon can destabilize a compound by reducing the α-carbon electron density, with the effect being more notable on sp^3^ α-carbons than sp^2^.^20^ This suggests that TAs are more susceptible to the electron-withdrawing effects of substituents on the α-carbon than unsaturated compounds, specifically the unreacted forms of Raman probes. Thus, we hypothesized that replacing esters with less electron-withdrawing amides could reduce the destabilizing effect on sp^3^ α-carbons of the TA, thereby increasing the TA ratio. To test this hypothesis, thia-Michael reactions of ThioRas and the amide derivative iPrCAA with methanethiol were analyzed using density functional theory (DFT) calculations (Figure 1d). The resulting TA formed a mixture of two diastereomers. The Δ*G* values for ThioRas and iPrCAA (averaged Δ*G* values indicated in red in the figure) were +0.2 and −0.8, respectively, supporting the hypothesis that the amide derivative can promote adduct formation.

### Reaction Dynamics and Structure–Activity Relationships

The rapid equilibrium and reversibility of iPrCAA were confirmed through a reaction with 2-mercaptoethanol (Figure 2).^21^ Measurements were obtained in a 3:1 mixture of dimethyl sulfoxide (DMSO) and phosphate-buffered saline (PBS). When 1.1 equiv of 2-mercaptoethanol was added to 90 mM iPrCAA, the Raman spectrum measured after 5 min showed peaks at 2231 cm^–1^ for iPrCAA and 2247 cm^–1^ for the TA. The molar ratio, adjusted for relative Raman intensity (Figure S1), was 8:92 (i), which remained unchanged after 30 min, confirming rapid equilibrium (ii). Diluting the solution 3-fold and 10-fold shifted the ratios to 14:86 and 22:78, respectively, demonstrating reversibility (iii, iv). Proton nuclear magnetic resonance (NMR) data further corroborated these results, aligning closely with the Raman data (Figure S2).

**Figure 2:**
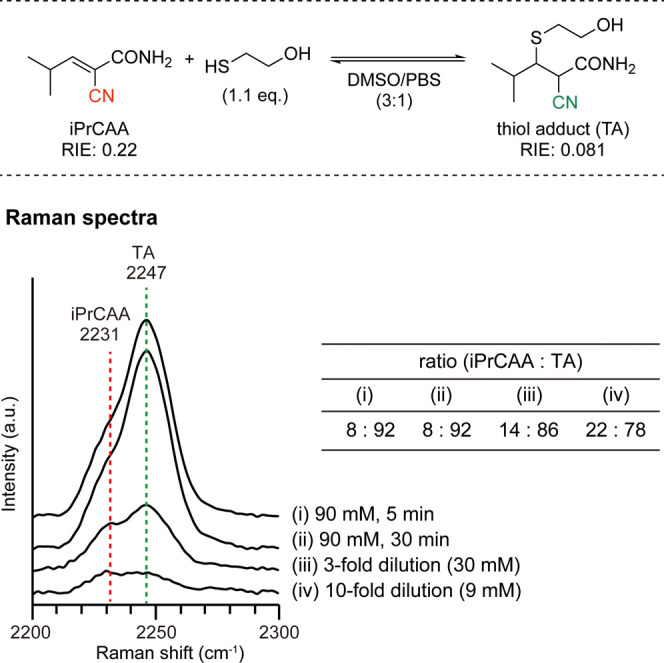
Confirmation of rapid equilibrium and reversibility in the thia-Michael reaction of iPrCAA and 2-mercaptoethanol in DMSO/PBS (3:1). Dilution was performed without altering the DMSO concentration. Ratios were determined from the peak areas of the Raman spectra. DMSO, dimethyl sulfoxide; PBS, phosphate-buffered saline; a.u., arbitrary units; eq, equivalents; (*E*)-2-cyano-3-isopropylacrylamide, iPrCAA.

In addition to ThioRas and iPrCAA, amide derivatives with varying *R* substituents were synthesized to assess the effect of the substituent on the thia-Michael reaction ratios (Figure S3). Figure 3 presents the results for 9 mM (where differences between the compounds were more pronounced) as a subset of the data shown in Figure S3. To simplify the DFT analysis, Δ*G* values were calculated by using methanethiol instead of 2-mercaptoethanol. The proportions of CAAs and their TAs were measured using 1.1 equiv of 2-mercaptoethanol, as in the spectra shown in Figure 2. Raman analysis confirmed that adduct ratios decreased with dilution, demonstrating the reaction reversibility (Figure S3). iPrCAA exhibited a higher adduct ratio (22:78) than ThioRas (42:58), attributable to its stabilizing effect on TAs. Derivatives with cyclohexyl or phenyl groups also resulted in the formation of the adduct with a higher concentration. The electron-donating group on the phenyl ring of 4OMeCAA stabilized the electron-deficient CAA alkene, hindering adduct formation (49:51). Introducing a single halogen atom (2FCAA and 4BrCAA) resulted in adduct ratios comparable to those of 2-cyano-3-phenylacrylamide (PhCAA), whereas the strong electron-withdrawing substituent CF_3_ destabilized the CAA alkene, promoting adduct formation (35CF_3_CAA, 7:93). These ratios were consistent with the trends predicted from the Δ*G* values.

**Figure 3:**
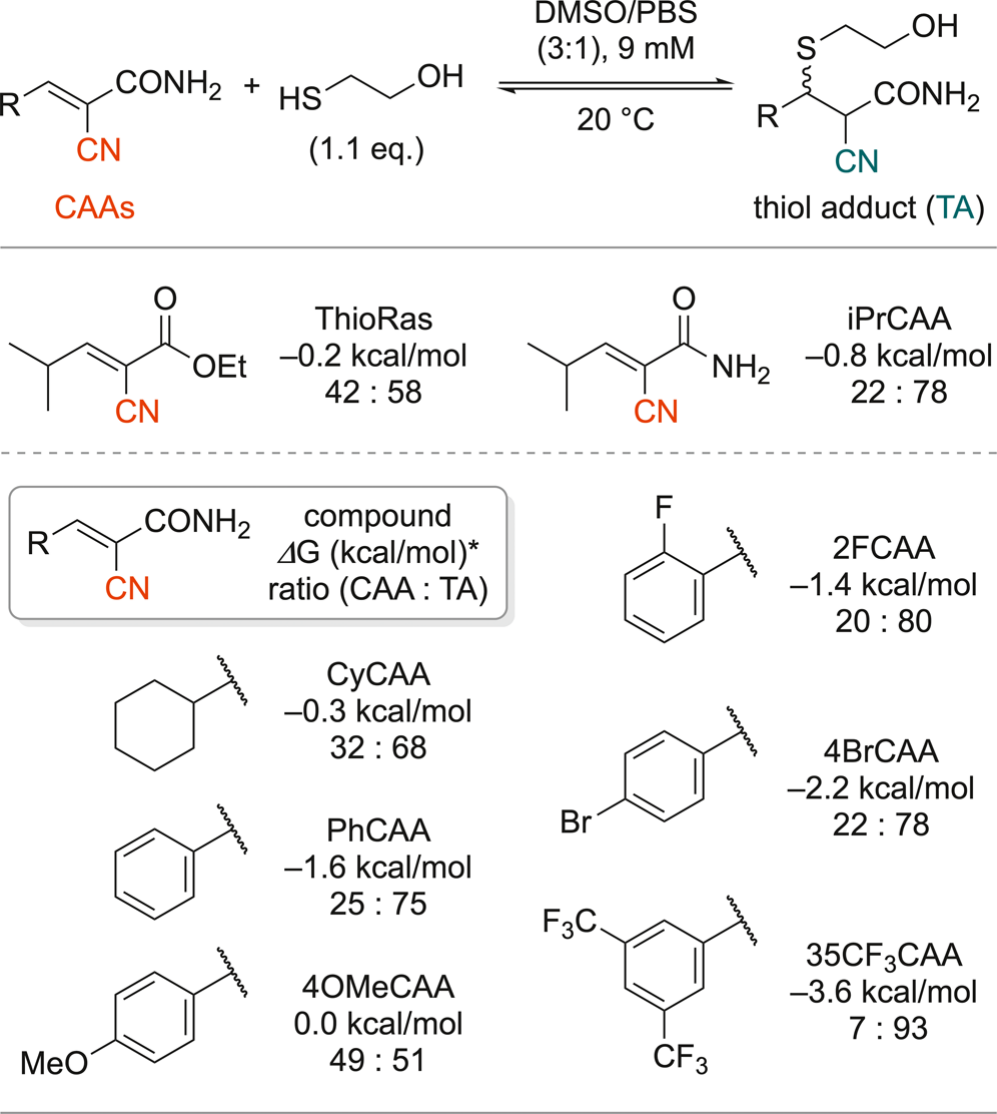
Thia-Michael reaction of 2-cyanoacrylamides with 2-mercaptoethanol. *For calculations, MeSH was used instead of mercaptoethanol. Δ*G* was computed at the M06-2X/6-311+G­(d,p) level of theory.

### Sensitivity Enhancement and Chemical Selectivity

The solubility of iPrCAA in water was investigated. DMSO in concentrations of 0.1–2% is typically used when introducing Raman probes into cells. However, high concentrations can exert cytotoxic effects and reduce the signal-to-noise ratio in Raman imaging. Therefore, improving the water solubility of the probe is preferable to avoid the use of DMSO. Amides, owing to their polarized structure, are generally less lipophilic than esters.^22^ Indeed, iPrCAA has a calculated partition coefficient (*C* Log *P* = 0.3422) lower than that of ThioRas (2.186) (Figure S1). The water solubility of iPrCAA was evaluated on the basis of turbidity assessments (Figures 4a and S4). ThioRas exhibited turbidity at concentrations of >3 mM and formed a suspension at 30 mM (blue). In contrast, iPrCAA remained fully dissolved at 30 mM (red). Among the amide derivatives, those with aromatic rings exhibited higher hydrophobicity and crystallized more easily, leading to precipitation at lower concentrations (green). Based on these considerations, the compact amide derivative, iPrCAA, was identified as the most suitable candidate for the probe.

**Figure 4:**
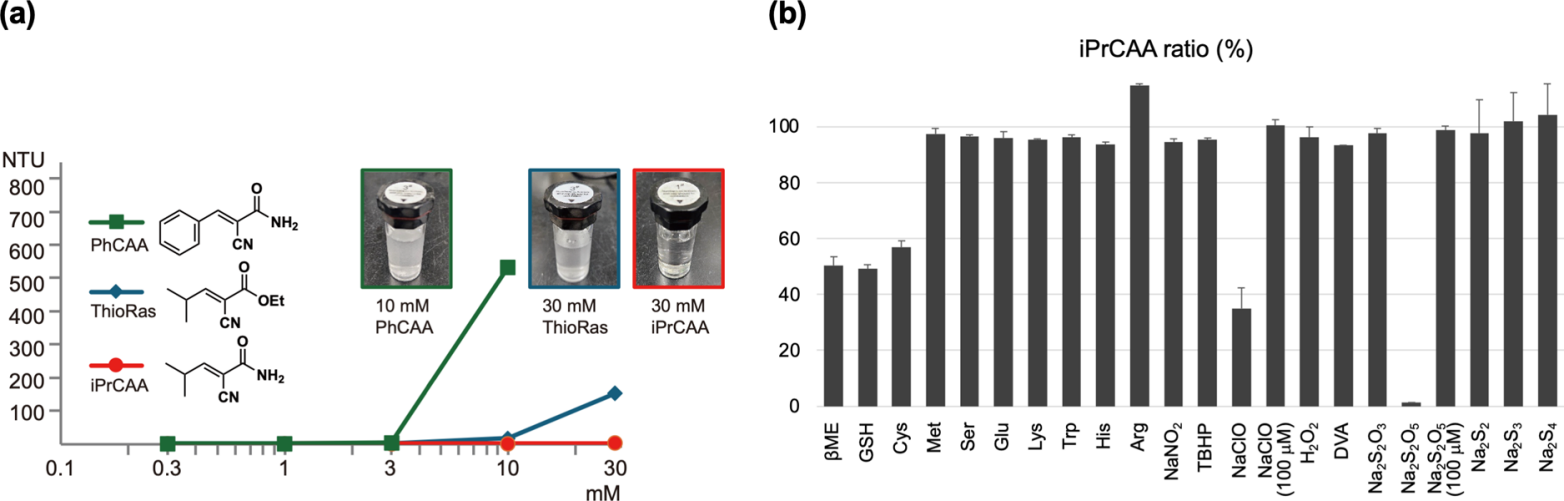
Evaluation of water solubility and chemical selectivity. (a) Solubility test in water based on turbidity assessments. Nephelometric turbidity units (NTUs) represent the turbidity index. Images within the box show solutions of 10 mM 2-cyano-3-phenylacrylamide (PhCAA, green), 30 mM ThioRas (blue), and (*E*)-2-cyano-3-isopropylacrylamide (iPrCAA, red). (b) Evaluation of the chemical selectivity of iPrCAA. Ten mM iPrCAA was incubated with 10 mM compounds in H_2_O, and iPrCAA ratios were determined from the height of the nitrile peaks in the Raman spectra. Glutathione (GSH) was neutralized using an aqueous sodium bicarbonate solution. Owing to solubility, 50% aqueous DMSO was used for DVA ((±)-1,2-dithiolane-3-valeric acid). For neutralization, 50 mM 3-(cyclohexylamino)-1-propanesulfonic acid (CAPS) buffer was used for Na_2_S_2_, Na_2_S_3_, and Na_2_S_4_. βME, 2-mercaptoethanol; Cys, l-cysteine; Met, l-methionine; Ser, l-serine; Glu, l-glutamic acid; Lys, l-lysine; Trp, l-tryptophan; His, l-histidine; Arg, l-arginine; TBHP, *tert*-butyl hydroperoxide.

Given the water solubility of iPrCAA, its thia-Michael reaction with 2-mercaptoethanol was analyzed by deriving Raman spectra in water without DMSO (Figure S5). At a concentration of 9 mM, the iPrCAA:TA ratio was 26:74, comparable to that observed in the presence of DMSO.

The chemical selectivity of iPrCAA was evaluated using nucleophilic amino acids, biologically relevant oxidants, and sulfur species, including supersulfides, all tested at 10 mM to assess potential off-target reactivity (Figure 4b). As expected, the representative thiols 2-mercaptoethanol (βME), glutathione (GSH), and l-cysteine (Cys) reacted with iPrCAA to form adducts. Although most tested compounds showed no detectable reactivity, Na_2_S_2_O_5_ and NaClO reacted with iPrCAA. Na_2_S_2_O_5_ irreversibly formed a sulfonic acid adduct via conjugate addition of sulfite,^23^ whereas NaClO caused oxidative degradation of the probe.^24^ Nevertheless, neither compound showed any reactivity at 100 μM, which is considered to be the upper limit of physiologically relevant concentrations. These results suggest that iPrCAA enables the quantification of intracellular thiols with minimal interference from other biologically relevant species under physiological conditions.

### Live-Cell Imaging

Motivated by these promising results, we proceeded with the quantification of the endogenous thiols. Human cervical cancer (HeLa) cells were treated with 8 mM iPrCAA, and Raman analysis was performed. No apparent cytotoxicity was observed under these high-concentration conditions during Raman imaging. At this concentration, ThioRas aggregates in the medium without the addition of glutathione,^13^ highlighting the importance of the aqueous solubility of iPrCAA. Figure 5a shows the iPrCAA and TA concentrations, estimated from peak intensities using water as an internal standard. The averaged Raman spectra of the medium, cytoplasm, nucleoplasm, nucleolus, and lipid droplets (LDs) are shown in Figure 5b. The black line represents the original spectra, whereas the red and green lines indicate the fitted nitrile peaks of iPrCAA and TAs, respectively. Nitrile peaks exhibited partial overlap but were separable, as expected. Probe concentrations (sum of iPrCAA and TA) in the cytoplasm, nucleoplasm, nucleolus, and LDs were 13.2, 14.3, 17.2, and 11.8 mM, respectively, slightly higher than the concentration in the medium (7.1 mM). ThioRas has been demonstrated to preferentially accumulate in LDs.^13^ In contrast, water-soluble iPrCAA exhibited a more uniform distribution. Thiol concentrations were estimated using a calibration curve based on the spectra measured from individual solutions (Figure S6). No TA peaks were detected in the medium. Thiol concentrations of 8.7, 10.8, 12.8, and 5.3 mM were observed in the cytoplasm, nucleoplasm, nucleolus, and LDs, respectively, slightly higher than the reported thiol concentrations (primarily glutathione) in HeLa cells (∼5 mM).^22,25–28^ The probe and thiol concentrations were visualized through Raman images constructed based on the intensities of two nitrile peaks and their ratio (Figure 5c). Although iPrCAA itself was uniformly distributed, regions with high TA concentrations tended to exhibit elevated thiol levels. Accordingly, the slight accumulation of the probe in the cells was attributed to its adduct formation with endogenous thiols. Furthermore, a comparison with the distribution of endogenous cellular CH stretching (lipids: 2825–2995 cm^–1^), phenyl groups (proteins: 999–1016 cm^–1^), phosphate (DNA and RNA: 777–802 cm^–1^), and cytochrome *c* (mitochondria: 734–772 cm^–1^) revealed that thiols were distributed similarly to proteins, likely due to the presence of cysteine residues in the proteins (Figure S7a). This ability to visualize the spatial distribution of multiple unlabeled endogenous molecules reflects a unique advantage of Raman imaging, offering a complementary approach to fluorescence-based techniques that rely on specific labeling.

**Figure 5:**
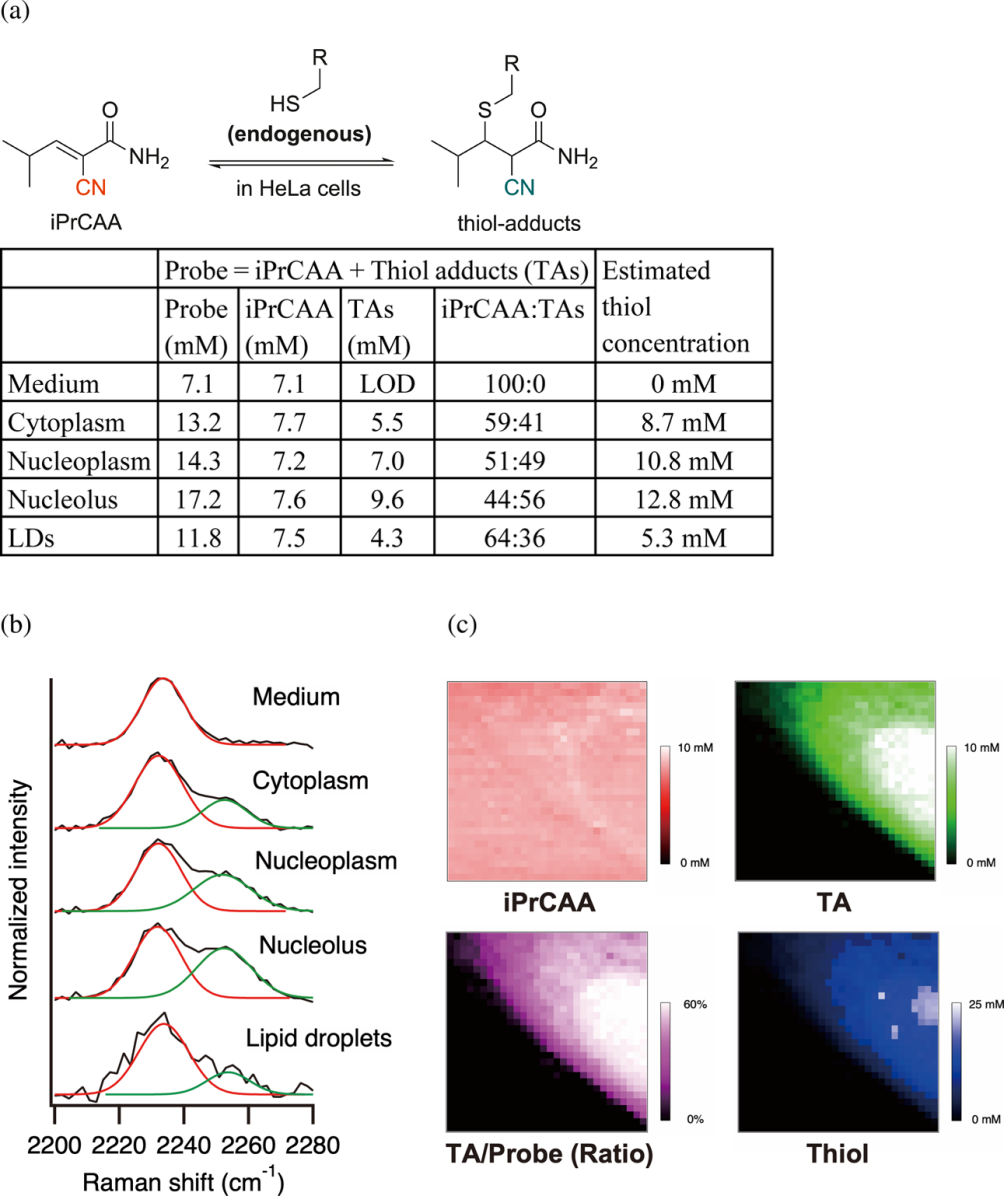
Quantitative analysis of endogenous thiols after exposure to 8 mM iPrCAA in HeLa cells. The concentrations of iPrCAA and its TAs were calculated from the ratio of the peak intensities of iPrCAA and water. (a) Ratio of iPrCAA to TAs and estimated concentrations of the probe and endogenous thiol. (b) Averaged Raman spectra. (c) Raman images of the concentration distributions of iPrCAA, TA, thiol, and the ratio of TA to iPrCAA constructed based on the peak intensity of the nitriles. Although there is a shift of 1–2 cm^–1^ depending on the region, the Raman shifts of iPrCAA and TA are approximately 2232 cm^–1^ and 2252 cm^–1^, respectively. LDs, lipid droplets; LOD, below the limit of detection; (*E*)-2-cyano-3-isopropylacrylamide, iPrCAA.

When the iPrCAA concentration was reduced to 4 and 2 mM, the TA proportion increased concomitantly with the decrease in concentration (Figure S7bc). In contrast, the estimated intracellular thiol concentration remained constant regardless of the probe concentration, highlighting the robustness of this quantification method. Additionally, analysis of 41 cells revealed that thiol concentrations varied by several-fold among individual cells (Figure S8).

Finally, to evaluate whether the proposed method could detect responses to biological stimuli, we assessed the effect of switching to a thiol-free medium on the endogenous thiol concentration in living cells (Figures 6 and S9 and S10). Following the medium exchange, the thiol concentration decreased over time. After 4 h, the concentration decreased to approximately two-thirds of its pretreatment level. By 48 h, the concentration reached a nearly steady state, converging to approximately 4 mM across intracellular locations. For LDs, no data with a sufficient signal-to-noise ratio for quantification could be obtained. We compared the distribution of intracellular thiol concentrations before and after treatment with a thiol-free medium. By 48 h post-treatment, the variation across locations had diminished, resulting in almost uniform contrast (Figures 6b and S10). This depletion effect was assessed in live cells, representing a pioneering analysis in this context.

**Figure 6:**
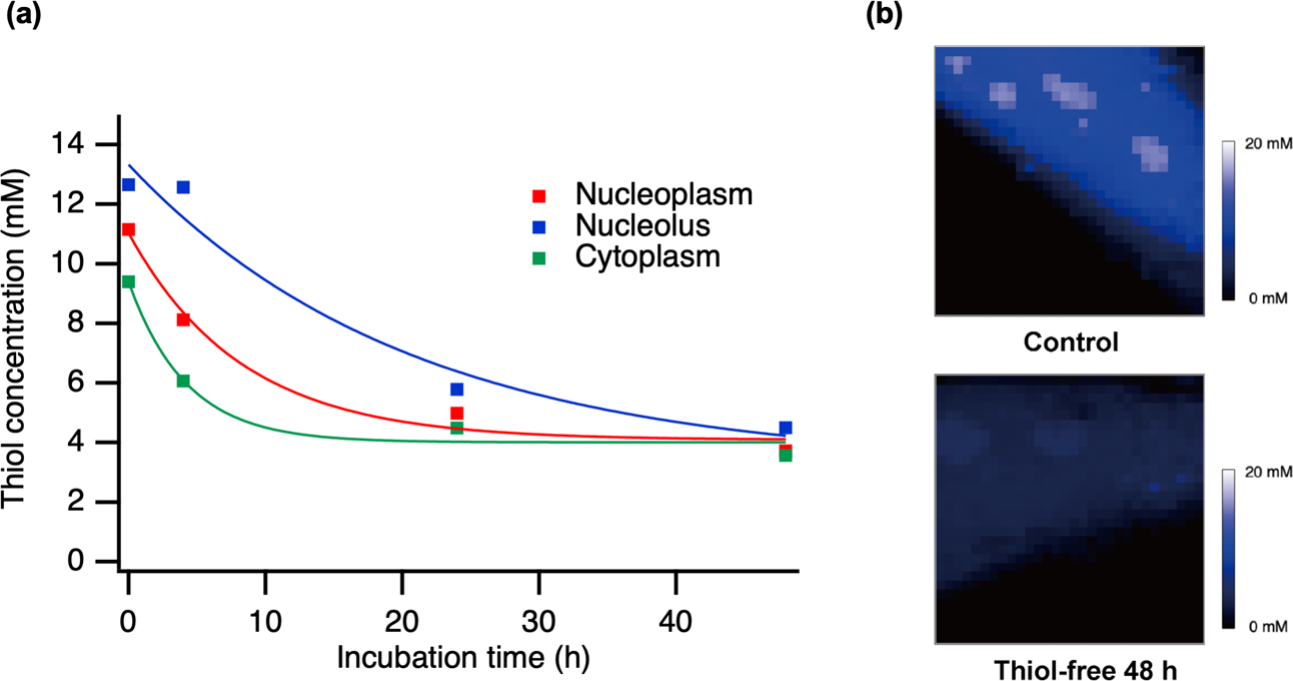
Quantification of intracellular thiols following replacement with a thiol-free medium in HeLa cells. (a) Time-course quantification of thiol concentration using 4 mM iPrCAA. (b) Raman image of thiol concentration distribution with 8 mM iPrCAA. Above: untreated control; below: 48 h treatment with a thiol-free medium. Color indicates thiol concentration (mM). LOD, below the limit of detection; (*E*)-2-cyano-3-isopropylacrylamide, iPrCAA; TA, thiol adduct.

## Conclusion

This study represents the first successful quantification of endogenous thiols using Raman probes. Accurate intracellular thiol quantification remains a significant challenge, despite its critical role in biological processes.^29–32^ Existing quantification methods can be broadly categorized into separation-based techniques, such as high-performance liquid chromatography and in situ detection methods using fluorescent probes.^33^ Although fluorescent probes are highly sensitive, signal saturation poses a challenge when quantifying substances at high concentrations such as intracellular thiols. Ratiometric fluorescent probes based on reversible reactions can mitigate this issue and enable real-time and spatially resolved measurements.^28^ However, fluorescent probes often exhibit uneven intracellular distribution owing to the bulky nature of their dyes. In comparison, Raman probes with compact tags provide a more uniform intracellular distribution. Nevertheless, the existing probe, ThioRas, could not detect endogenous thiols.^13^ The iPrCAA probe that we developed addresses this limitation through optimized reactivity and solubility. Notably, live-cell quantification using Raman probes is more straightforward than with fluorescent probes,^22,25–27^ as water can serve as a concentration standard.^15,16,34^ In addition, Raman imaging facilitates a comparison of the spatial distribution of various endogenous molecules, enabling multiparametric analysis without the need for labeling. The cyanoacrylic acid scaffold serves as an excellent platform for thiol detection in both fluorescence and Raman spectroscopy. However, it exhibits certain limitations. Intracellular thiols include various species, such as cysteine, glutathione, and protein thiols, and the reaction efficiency with probes varies depending on the thiol species and the surrounding environment. Each existing method for thiol quantification, such as fluorescent probes and mass spectrometry, has its own advantages and disadvantages, and cross-validating results using different techniques is currently considered to be important.^35,36^ Therefore, further advancements in probe design and analytical methodologies are critical to achieving precise and reliable thiol quantification.

Recent advancements in live-cell Raman imaging have increasingly focused on enhancing practicality by leveraging nonlinear Raman techniques, such as stimulated Raman scattering.^2,3,37–39^ By exploiting the high spectral resolution of Raman probes, multiplex dye palettes have been developed, which can enable multiplex staining with over 20 different dyes.^39^ These advancements serve as a valuable complement to fluorescent probes. However, the potential benefits of compact Raman probe development remain underexplored, likely owing to structural constraints associated with the need for enhanced sensitivity. Typically, achieving resonance effects necessitates a conjugated structure comparable in size to that of fluorescent dyes, contributing to larger probe designs. This study highlights the potential of compact Raman probes, exemplified by iPrCAA (molecular weight: 138 g/mol). The quantification of thiol concentrations in blood and cells is closely associated with oxidative stress and has been investigated as a potential diagnostic marker and prognostic indicator for various diseases.^29,40–42^ The complementary development of Raman and fluorescence probes targeting thiol quantification significantly enhances this critical analysis. In this study, iPrCAA was utilized in a cellular system; however, its potential advantages for in vivo applications have not yet been established. This remains a key limitation of this study and an important subject for future investigation. Future studies will therefore focus on exploring the unique characteristics and potential applications of these probes, particularly in conjunction with the enhanced sensitivity enabled by nonlinear Raman scattering phenomena.

## Supplementary Material



## Notes

Computational study, Raman shift, experimental procedures including characterization data, copies of ^1^H and ^13^C NMR spectra, and additional references (PDF)

## Abbreviations

βME: 2-mercaptoethanol
CAA: 2-cyanoacrylamide
CAPS: 3-(cyclohexylamino)-1-propanesulfonic acid
CYS: l-cysteine
DFT: density functional theory
DMSO: dimethyl sulfoxide
FCCP: carbonyl cyanide *p*-trifluoromethoxyphenylhydrazone
GSH: glutathione
HBSS: Hank’s balanced salt solution
iPrCAA: (*E*)-2-cyano-3-isopropylacrylamide
LD: lipid droplet
LOD: below the limit of detection
NMR: nuclear magnetic resonance
NTU: nephelometric turbidity unit
PBS: phosphate-buffered saline
PhCAA: 2-cyano-3-phenylacrylamide
RIE: relative intensity enhancement
SVD: singular-value decomposition
TA: thiol adduct
