# Supplementary material for: Visualizing Thiol Stress Responses in Cells with a Water-Soluble Raman Sensor
Source: Anal Chem. 2025 Nov 10;97(46):25398–405. doi: 10.1021/acs.analchem.5c04391 (PMC12658863; doi:10.1021/acs.analchem.5c04391)
Supplement: Supplementary file 1 [file ac5c04391_si_001.pdf]

Supporting Information

Visualizing Thiol Stress Responses in Cells with a Water-Soluble Raman Sensor

Hiroyuki Yamakoshi,*[1] Meichen Wang,[1] Keisuke Koga,[1] Shinji Kajimoto,[1] Yuse Kuriyama,[1] Yusuke Sasano,[1] Takaaki Akaike,[2] Yoshiharu Iwabuchi,[1] and Takakazu Nakabayashi[1]

[1]Graduate School of Pharmaceutical Sciences, Tohoku University, 6-3 Aoba, Aramaki, Aoba-ku, Sendai 980-8578, Japan.
[2]Department of Environmental Medicine and Molecular Toxicology, Graduate School of Medicine, Tohoku University, 2-1 Seiryo-machi, Aoba-ku, Sendai, 980-8575, Japan

*Corresponding author E-mail: hiroyuki.yamakoshi.e1@tohoku.ac.jp

Table of Contents

1. Supplementary Figures (**Figure S1–S10**) S2–S12
2. Computational Study S13–S42
3. Raman Shift (**Table S1**) S43
4. Experimental Procedures (**Scheme S1**) S44–S48
5. Copies of ^{1}H and ^{13}C NMR Spectra (**Figure S11–S24**) S49–S55
6. Additional References S56

1. Supplementary Figures

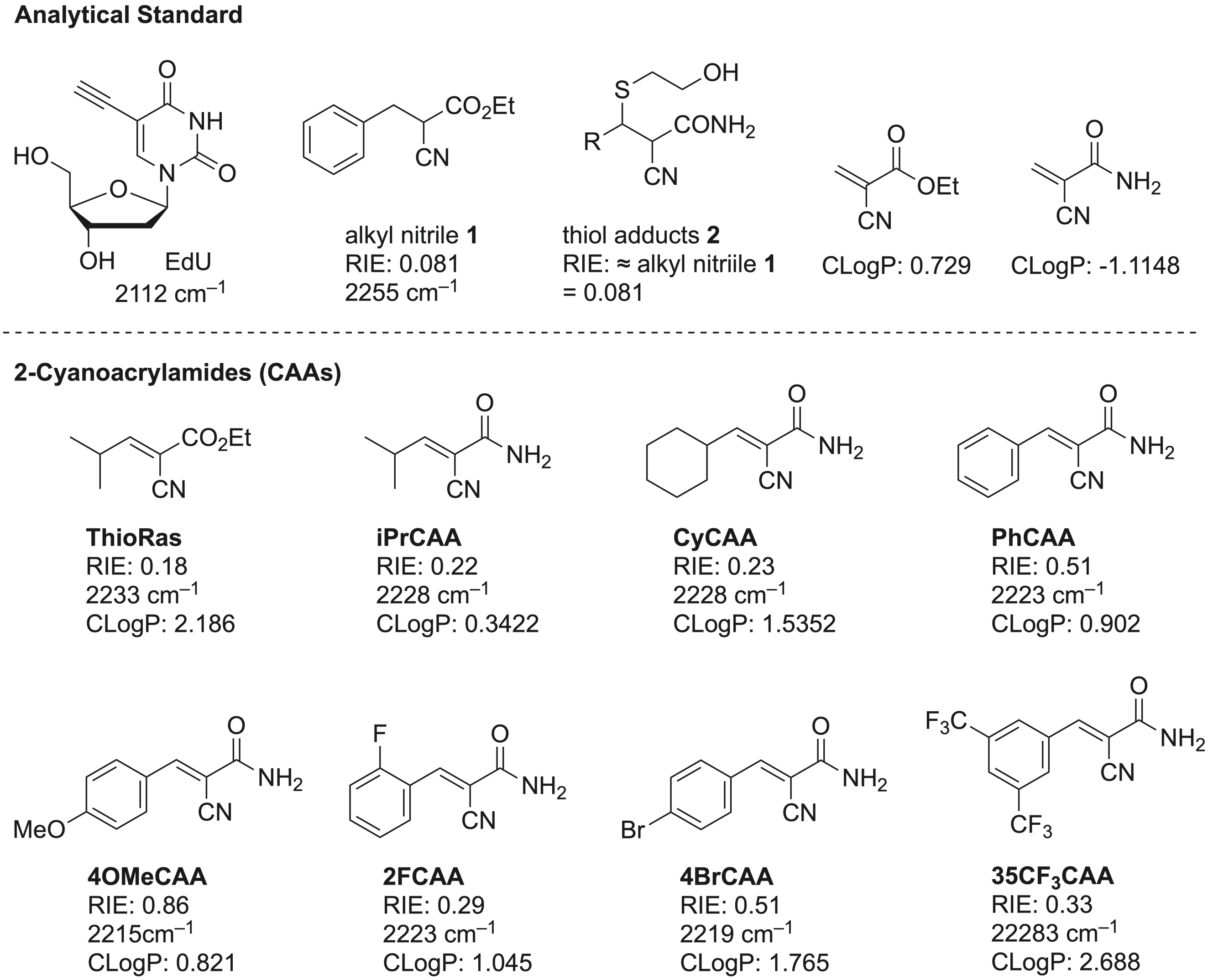

Figure S1. Relative Raman intensity vs. 5-ethynyl-2'-deoxyuridine (EdU) (RIE) values of nitriles. The laser wavelength was set as 532 nm. Based on prior work (*J. Am. Chem. Soc.* **2012**, *134*, 20681)[1], the RIE values were calculated based on the ratio of peak areas obtained from mixtures of nitriles and EdU diluted in dimethyl sulfoxide. The RIE values for thiol adducts **2** were approximated using that for alkyl nitrile 1 (0.081) owing to the inability to isolate them. CLogP values were calculated using ChemDraw (version 23.0.1).

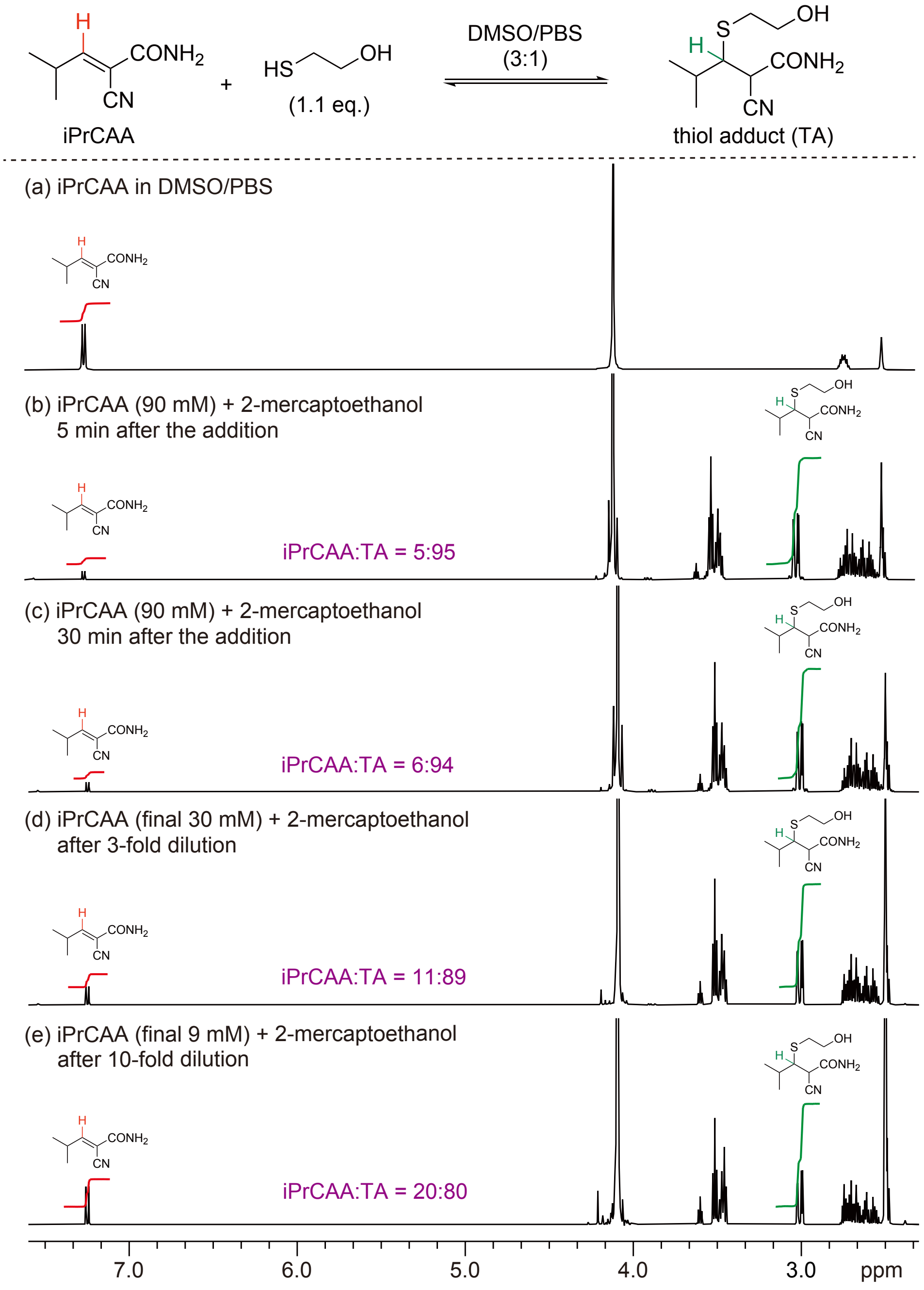

Figure S2. ^{1}H-NMR analysis of the thia-Michael reaction. (a) ^{1}H-NMR spectrum of iPrCAA in DMSO-d_6/PBS-*d*. (b) iPrCAA was treated with 1.1 equivalents of 2-mercaptoethanol. After 5 min, a 5:95 mixture of iPrCAA and TA was obtained. (c) After 30 min, the iPrCAA:TA ratio (6:94) remained unchanged, indicating that the thia-Michael reaction was completed within 5 min. (d, e) Upon 3- or 10-fold dilution of the mixture (c), a higher proportion of iPrCAA was detected, which confirmed the reversible nature of the thia-Michael reaction with iPrCAA and 2-mercaptoethanol. NMR, nuclear magnetic resonance; DMSO, dimethyl sulfoxide; PBS, phosphate-buffered saline; iPrCAA, (*E*)-2-cyano-3-isopropylacrylamide; TA: thiol adduct

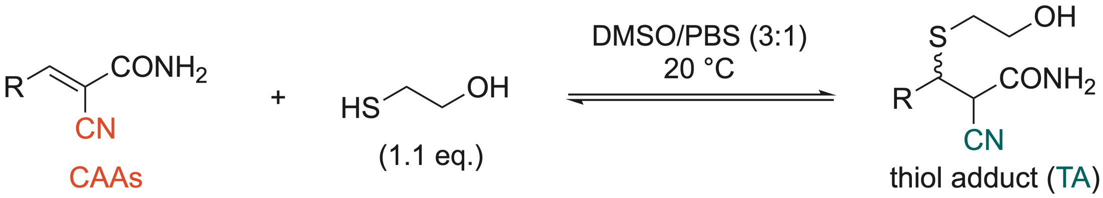

structure	name	ΔG (kcal/mol)*		concentration	ratio (CAA : TA) Raman	ratio (CAA : TA) ^{1}H-NMR
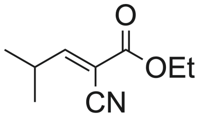	ThioRas	average	+0.2	90 mM	17 : 83	13 : 87
		(*syn*	–0.4	30 mM	28 : 72	20 : 80
		anti	+0.7)	9 mM	42 : 58	43 : 57
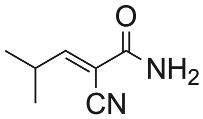	iPrCAA	average	–0.8	90 mM	8 : 92	6 : 94
		(*syn*	–0.8	30 mM	14 : 86	11 : 89
		anti	–0.8)	9 mM	22 : 78	20 : 80
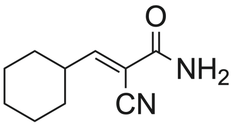	CyCAA	average	–0.3	90 mM	10 : 90	10 : 90
		(*syn*	–0.1	30 mM	20 : 80	18 : 82
		anti	–0.5)	9 mM	32 : 68	25 : 75
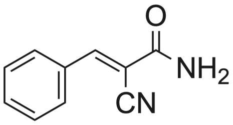	PhCAA	average	–1.6	90 mM	11 : 89	7 : 93
		(*syn*	–1.3	30 mM	17 : 83	15 : 85
		anti	–1.8)	9 mM	25 : 75	21 : 79
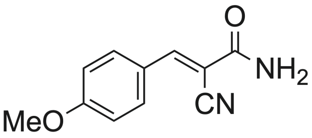	4OMeCAA	average	0.0	90 mM	23 : 77	18 : 82
		(*syn*	+0.2	30 mM	41 : 59	29 : 71
		anti	–0.2)	9 mM	49 : 51	48 : 52
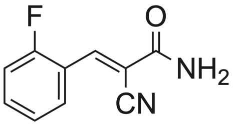	2FCAA	average	–1.4	90 mM	5 : 95	2 : 98
		(*syn*	–0.7	30 mM	11 : 89	5 : 95
		anti	–2.1)	9 mM	20 : 80	11 : 89
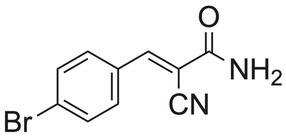	4BrCAA	average	–2.2	90 mM	10 : 90	5 : 95
		(*syn*	–2.1	30 mM	16 : 84	9 : 91
		anti	–2.2)	9 mM	22 : 78	16 : 84
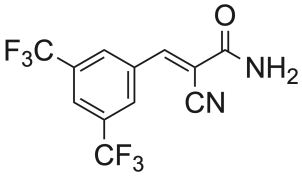	35CF$_3$CAA	average	–3.6	90 mM	2 : 98	2 : 98
		(*syn*	–3.1	30 mM	3 : 97	3 : 97
		anti	–4.0)	9 mM	7 : 93	7 : 93

Figure S3. Thia-Michael reaction of 2-cyanoacrylamides (CAAs) with 2-mercaptoethanol. *For calculations, MeSH was used instead of 2-mercaptoethanol. Δ*G* was computed at the M06-2X/6-311+G(d,p) level of theory. NMR, nuclear magnetic resonance; DMSO, dimethyl sulfoxide; PBS, phosphate-buffered saline

compound	0.3 mM	1 mM	3 mM	10 mM	30 mM
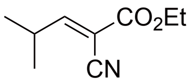 ThioRas	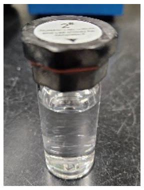 NTU 0.08	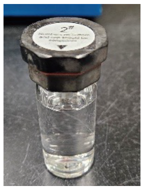 NTU 0.12	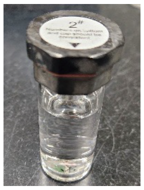 NTU 0.25	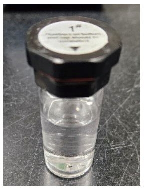 NTU 16.3	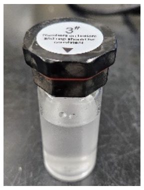 NTU 152
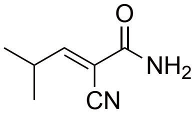 iPrCAA	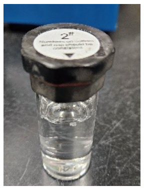 NTU 0.09	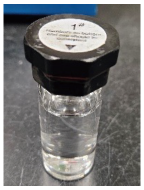 NTU 0.17	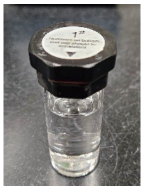 NTU 0.24	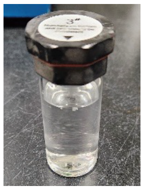 NTU 0.41	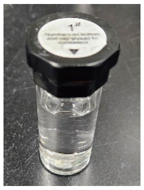 NTU 1.77
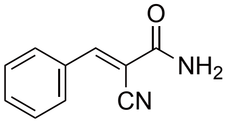 PhCAA	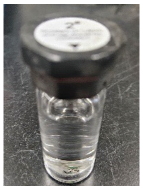 NTU 0.12	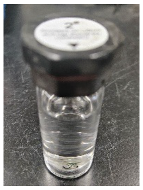 NTU 0.29	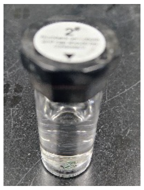 NTU 2.86	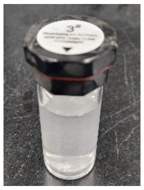 NTU 532	Analysis was not performed.
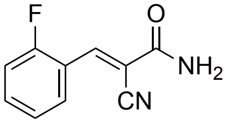 2FCAA	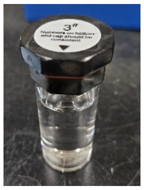 NTU 0.73	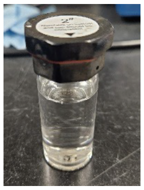 NTU 3.20	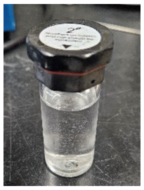 NTU 19.2	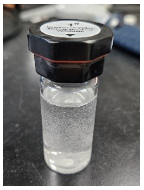 NTU 244	Analysis was not performed.

Figure S4. Turbidity assay of ThioRas and 2-cyanoacrylamides (CAAs) in water. Nephelometric turbidity units (NTU) represent the turbidity index. Although not shown in the figure, iPrCAA dissolved in water at a concentration of 100 mM. iPrCAA, (*E*)-2-cyano-3-isopropylacrylamide

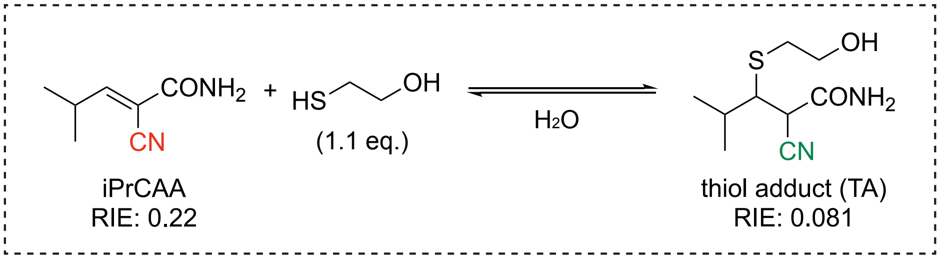

Raman spectra

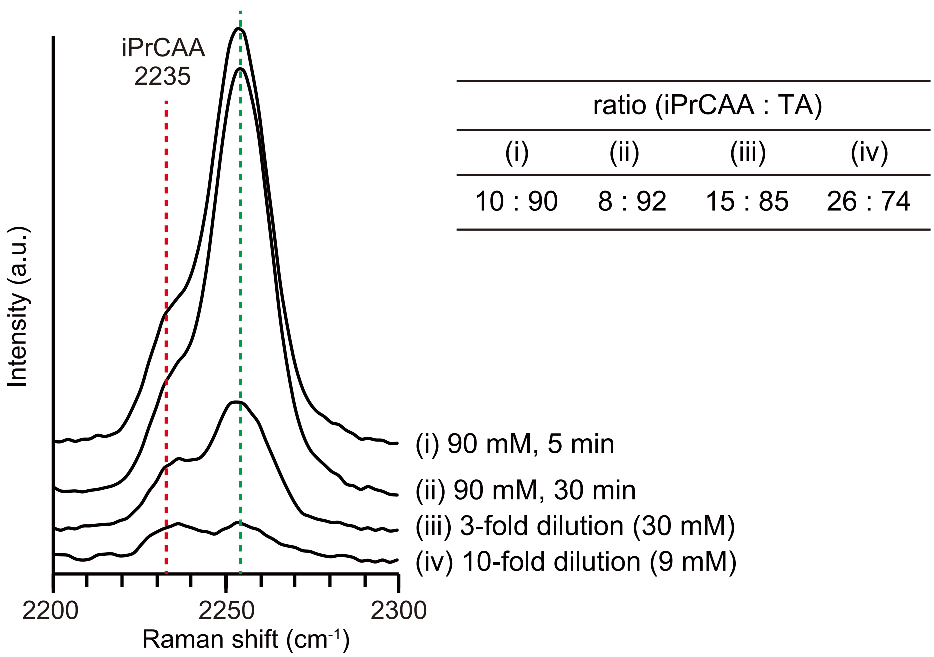

ratio (iPrCAA : TA)			
(i)	(ii)	(iii)	(iv)
10 : 90	8 : 92	15 : 85	26 : 74

Figure S5. Raman analysis of thia-Michael reaction in H_2O. iPrCAA and 2-mercaptoethanol were used in the reaction. Ratios were determined from the peak areas of the Raman spectra. a.u., arbitrary units; eq., equivalents. iPrCAA, (*E*)-2-cyano-3-isopropylacrylamide

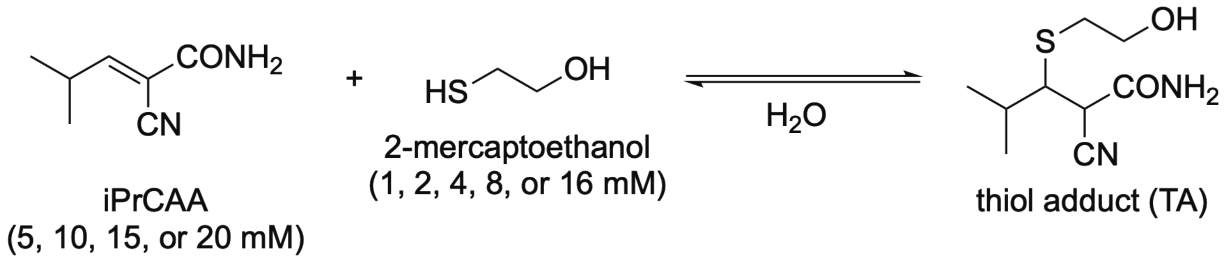

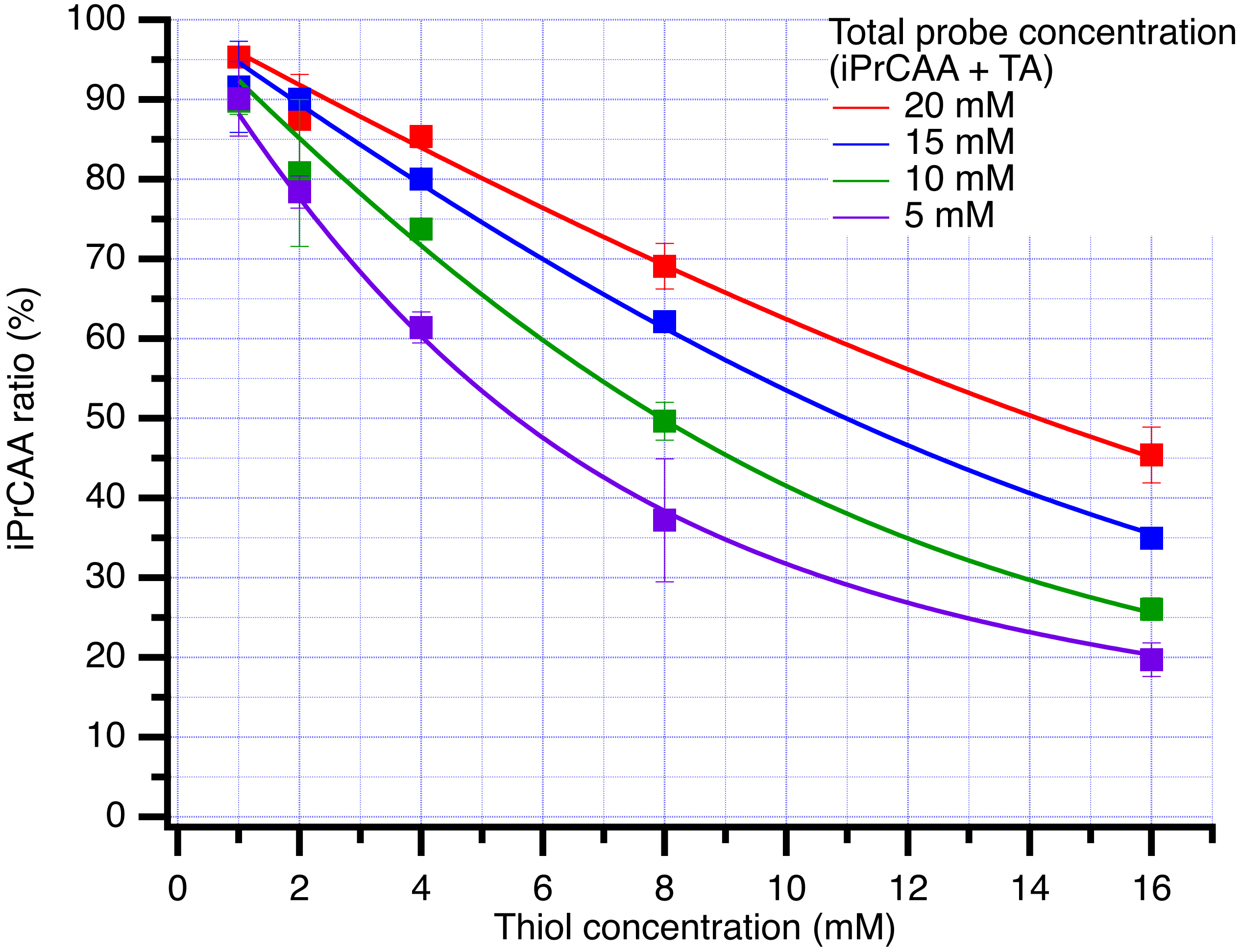

Figure S6. Thiol concentration calibration curve. iPrCAA (final concentration: 5, 10, 15, or 20 mM) and 2-mercaptoethanol (final concentration: 1, 2, 4, 8, or 16 mM) were mixed in H_2O. After 5 min, the Raman spectra of the reaction mixture were recorded. The iPrCAA concentration was calculated from the ratio of the peak intensities of iPrCAA and water. The concentration of the thiol-adduct was calculated by subtracting the iPrCAA concentration measured at each thiol concentration from the iPrCAA concentration measured without thiol. iPrCAA, (*E*)-2-cyano-3-isopropylacrylamide

(a) Quantitative analysis of endogenous thiols after exposure to **8 mM** iPrCAA

	Probe = iPrCAA + Thiol adducts (TAs)				Estimated thiol concentration
	Probe (mM)	iPrCAA (mM)	TAs (mM)	iPrCAA:TAs	
Medium	7.1	7.1	LOD	100:0	0 mM
Cytoplasm	13.2	7.7	5.5	59:41	8.7 mM
Nucleoplasm	14.3	7.2	7.0	51:49	10.8 mM
Nucleolus	17.2	7.6	9.6	44:56	12.8 mM
LDs	11.8	7.5	4.3	64:36	5.3 mM

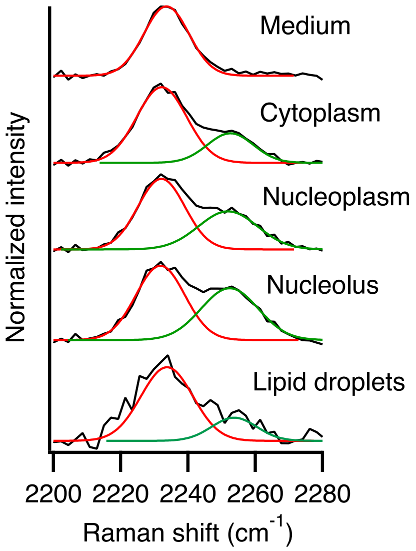

Raman images of HeLa cells after exposure to **8 mM** iPrCAA

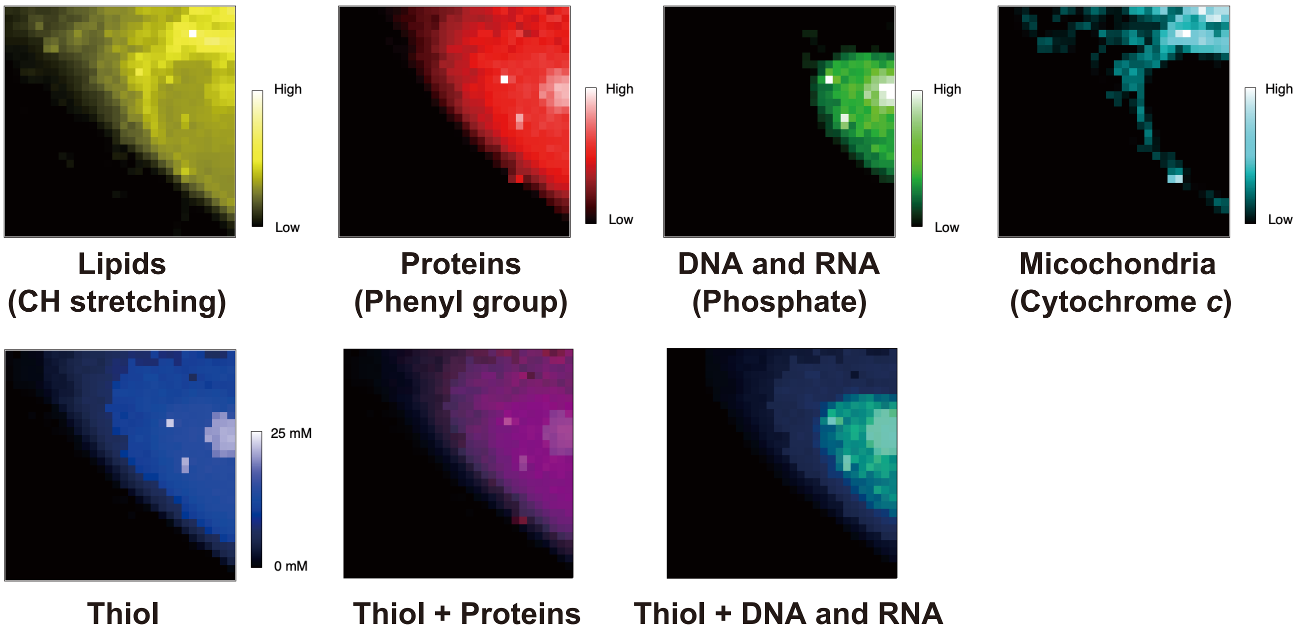

(b) Quantitative analysis of endogenous thiols after exposure to **4 mM** iPrCAA

	Probe = iPrCAA + Thiol adducts (TAs)				Estimated thiol concentration
	Probe (mM)	iPrCAA (mM)	TAs (mM)	iPrCAA:TAs	
Medium	3.4	3.4	LOD	100:0	0 mM
Cytoplasm	8.5	3.7	4.8	44:56	9.4 mM
Nucleoplasm	10.1	3.8	6.3	38:62	11.1 mM
Nucleolus	11.5	3.8	7.7	33:67	12.7 mM
LDs	–	LOP	LOP	–	–

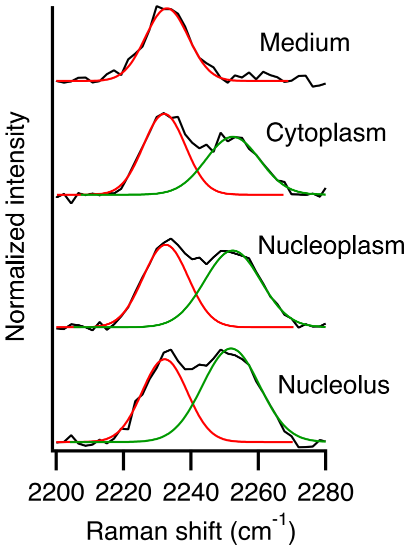

(c) Quantitative analysis of endogenous thiols after exposure to **2 mM** iPrCAA

	Probe = iPrCAA + Thiol adducts (TAs)				Estimated thiol concentration
	Probe (mM)	iPrCAA (mM)	TAs (mM)	iPrCAA:TAs	
Medium	1.4	1.4	LOD	100:0	0 mM
Cytoplasm	4.8	1.5	3.4	30:70	10.6 mM
Nucleoplasm	5.0	1.4	3.5	29:71	11.1 mM
Nucleolus	5.8	1.3	4.5	22:78	14.4 mM
LDs	–	LOP	LOP	–	–

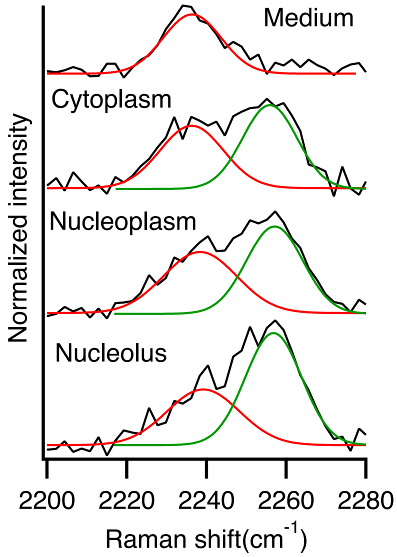

Figure S7. Quantitative analysis of endogenous thiols after exposure to (a) 8 mM iPrCAA, (b) 4 mM iPrCAA, and (c) 2 mM iPrCAA. The black line represents the original spectra, whereas the red and green lines indicate the fitted nitrile peaks of iPrCAA and TAs, respectively. HeLa cells were used. The concentration of iPrCAA and its thiol adducts was calculated from the ratio of the peak intensities of iPrCAA and water. The images were constructed based on the peak intensity of the nitriles, CH stretching (lipids: 2825–2995 cm^{-1}), phenyl groups (proteins: 999–1016 cm^{-1}), phosphate (DNA and RNA: 777–802 cm^{-1}), and cytochrome c (mitochondria: 734–772 cm^{-1}). While a shift of 1–2 cm^{-1} is observed depending on the region, the Raman shifts of iPrCAA and TA are approximately 2232 cm^{-1} and 2252 cm^{-1}, respectively. LDs, lipid droplets; LOD, below the limit of detection; LOP, below the limit of peak separation; (*E*)-2-cyano-3-isopropylacrylamide, iPrCAA.

(a)

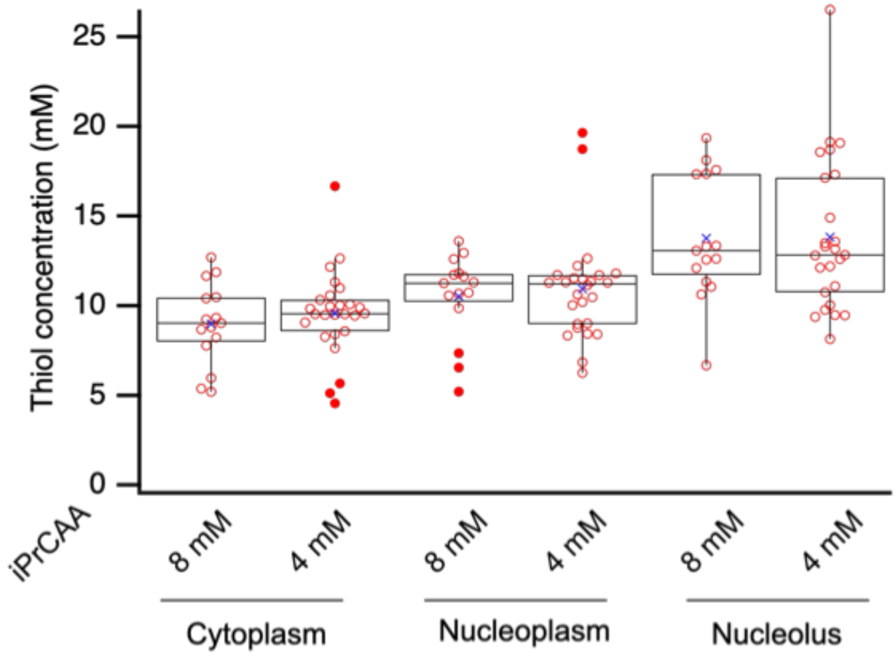

(b)

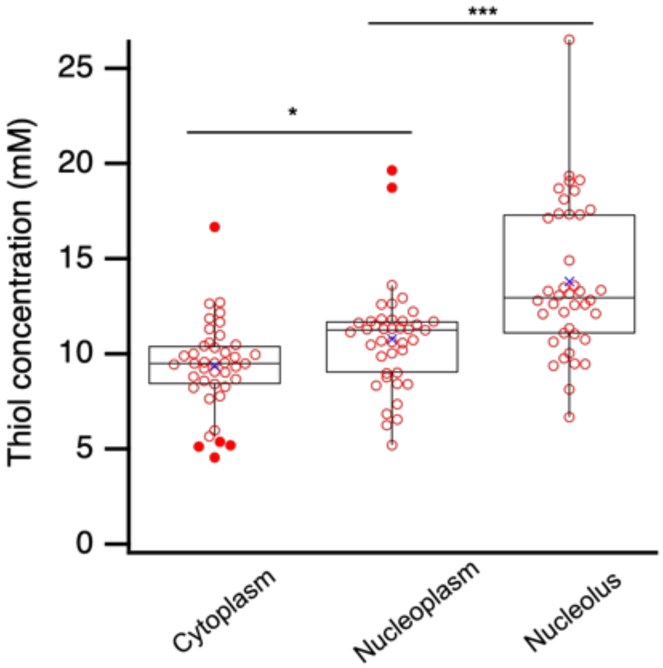

Figure S8. Quantification of thiol concentration in 41 cells treated with 4 mM or 8 mM iPrCAA. HeLa cells were used. (a) Concentration distribution distinguished between 4 mM (15 cells) and 8 mM (26 cells). (b) Combined concentration distribution for 4 mM and 8 mM conditions. Cross and solid circle markers represent averages and outliers, respectively. *: $p < 0.05$, ***: $p < 0.001$. n represents the number of cells measured.

(a) Quantification of intracellular thiols following replacement with thiol-free medium (**4 h**).

	Probe = iPrCAA + Thiol adducts (TAs)				Estimated thiol concentration
	Probe (mM)	iPrCAA (mM)	TAs (mM)	iPrCAA:TAs	
Medium	2.8	2.8	LOD	100:0	0 mM
Cytoplasm	6.2	2.9	3.3	47:53	6.1 mM
Nucleoplasm	7.2	2.7	4.5	38:62	8.1 mM
Nucleolus	8.1	2.7	5.4	33:67	12.6 mM

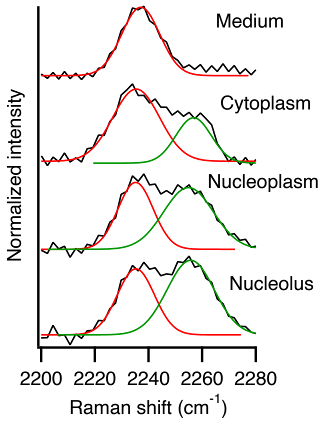

(b) Quantification of intracellular thiols following replacement with thiol-free medium (**24 h**).

	Probe = iPrCAA + Thiol adducts (TAs)				Estimated thiol concentration
	Probe (mM)	iPrCAA (mM)	TA (mM)	iPrCAA:TA	
Medium	2.9	2.9	LOD	100:0	0 mM
Cytoplasm	6.3	3.6	2.7	56:43	4.5 mM
Nucleoplasm	5.8	3.1	2.7	54:46	5.0 mM
Nucleolus	6.7	3.3	3.4	49:51	5.8 mM

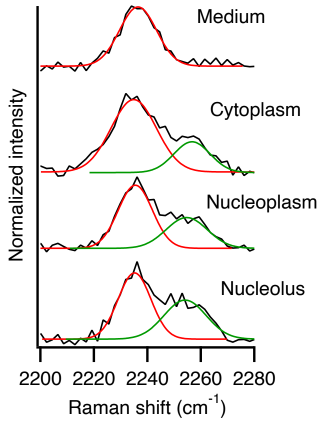

(c) Quantification of intracellular thiols following replacement with thiol-free medium (**48 h**).

	Probe = iPrCAA + Thiol adducts (TAs)				Estimated thiol concentration
	Probe (mM)	iPrCAA (mM)	TAs (mM)	iPrCAA:TAs	
Medium	3.4	3.4	LOD	100:0	0 mM
Cytoplasm	6.2	4.0	2.3	64:36	3.6 mM
Nucleoplasm	5.8	3.7	2.2	62:38	3.7 mM
Nucleolus	6.7	3.8	2.9	57:43	4.5 mM

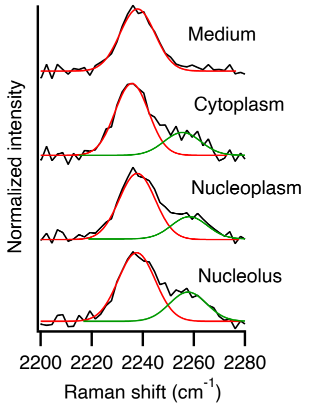

Figure S9. Quantification of intracellular thiols following replacement with a thiol-free medium using **4 mM iPrCAA**. HeLa cells were used. The black line represents the original spectra, whereas the red and green lines indicate the fitted nitrile peaks of iPrCAA and TAs, respectively. Data were obtained 4, 24, and 48 h after replacement [(a), (b), and (c), respectively]. LOD, below the limit of detection; (*E*)-2-cyano-3-isopropylacrylamide, iPrCAA.

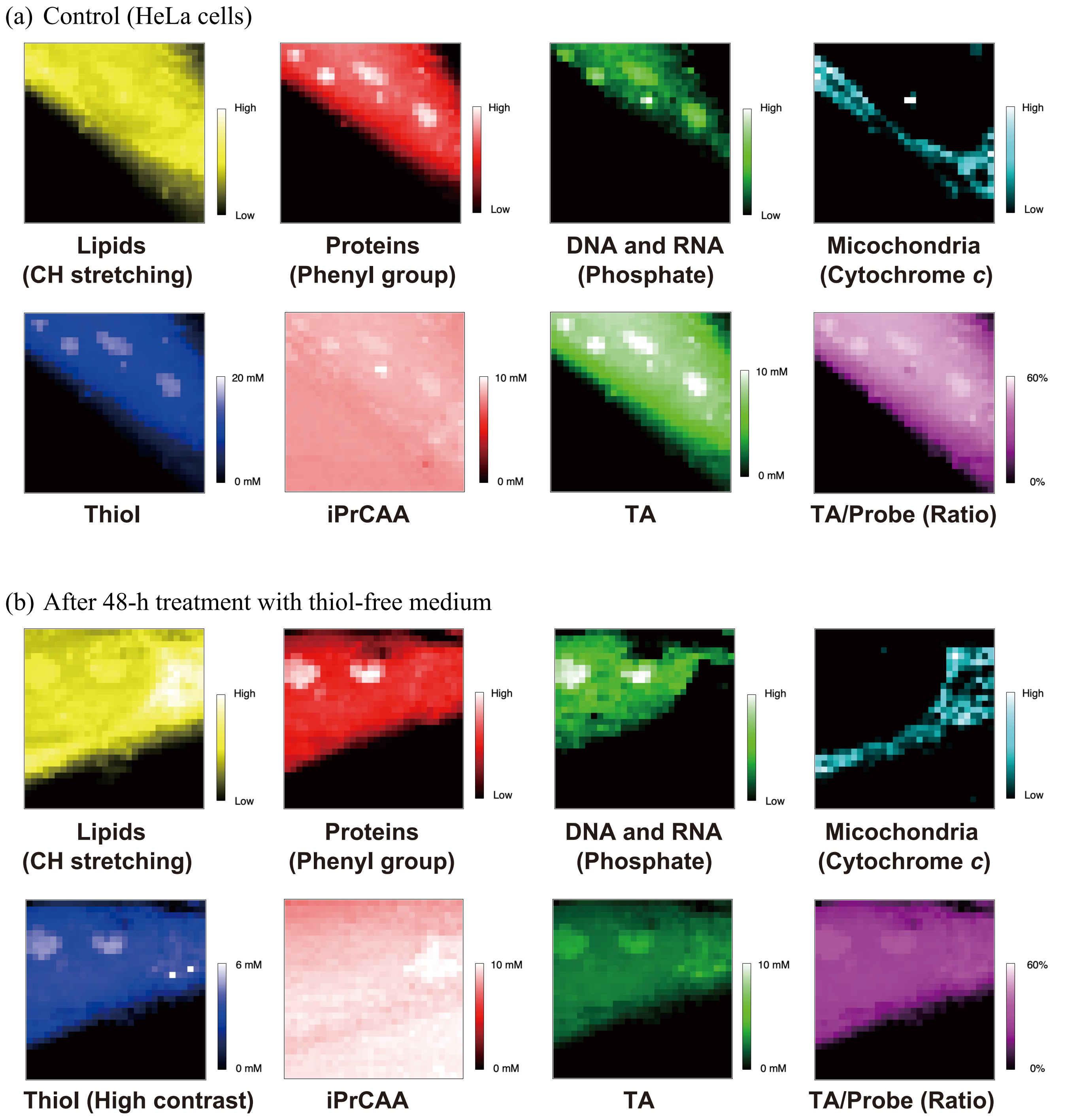

Figure S10. Raman images of intracellular thiols following replacement with a thiol-free medium, using **8 mM iPrCAA**. HeLa cells were used. (a) Raman images of control HeLa cells. (b) Raman images of HeLa cells after 48-h treatment with thiol-free medium. (*E*)-2-cyano-3-isopropylacrylamide, iPrCAA. The images were constructed based on the peak intensity of the nitriles, CH stretching (lipids: 2825–2995 cm^{-1}), phenyl groups (proteins: 999–1016 cm^{-1}), phosphate (DNA and RNA: 777–802 cm^{-1}), and cytochrome c (mitochondria: 734–772 cm^{-1})

2. Computational Study

Conformational searches were performed using Spartan ’20 (Wavefunction Inc.) with the Merk Molecular force field in the gas phase. Up to 500 conformers within 40 kJ/mol of the lowest-energy structure were retained. All recorded conformers for each molecule were further optimized at the DFT level with the B3LYP-D3 functional and 6-31G* basis set for all atoms. Single-point energy calculations in water were then carried out at the DFT level with the M06-2X functional and 6-311G(2d,p) basis set for all atoms.

Further DFT calculations were conducted for the most stable structure of each molecule, as obtained from the above conformational search, using Gaussian 16, Revision C.01 software. Geometry optimizations and frequency calculations were performed with the M06-2X functional and 6-311+G(d,p) basis set. Gibbs free energies (kcal/mol) were computed based on the energy and frequency calculations at the M06-2X/6-311+G(d,p) level. Solvent effects were accounted for using the self-consistent reaction field method with the conductor-like polarizable continuum model, employing water as the solvent. All stationary points were confirmed as local minima through frequency analysis.

The optimized coordinates, along with the corresponding energies

All energies are reported in Hartree.

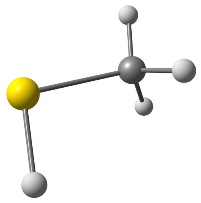

MeSH

Number of imaginary frequencies: 0

Optimized energy = –438.678981711

Sum of electronic and zero-point Energies = –438.632920

Sum of electronic and thermal Enthalpies = –438.628150

Sum of electronic and thermal Free Energies = –438.657660

H	1.281617000000	–0.835875000000	0.000000000000
S	–0.048357000000	–0.666049000000	0.000000000000
C	–0.048357000000	1.157319000000	0.000000000000
H	–1.092394000000	1.463448000000	0.000000000000
H	0.437318000000	1.542651000000	0.893017000000
H	0.437318000000	1.542651000000	–0.893017000000

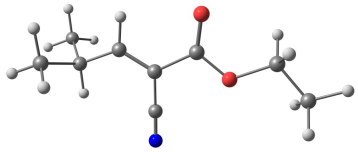

ThioRas

Number of imaginary frequencies: 0

Optimized energy = –555.901962848

Sum of electronic and zero-point Energies = –555.693469

Sum of electronic and thermal Enthalpies = –555.678712

Sum of electronic and thermal Free Energies = –555.735516

C	–3.564810000000	–0.278773000000	–1.263612000000
H	–3.697716000000	–1.363524000000	–1.289014000000
H	–3.036724000000	0.027323000000	–2.168128000000
H	–4.552328000000	0.186436000000	–1.263998000000
C	–2.799935000000	0.140538000000	0.000074000000
H	–2.677848000000	1.226819000000	0.000015000000
C	–3.564632000000	–0.278648000000	1.263907000000
H	–3.697544000000	–1.363394000000	1.289433000000
H	–4.552145000000	0.186571000000	1.264392000000
H	–3.036413000000	0.027530000000	2.168318000000
C	–1.458760000000	–0.510407000000	0.000010000000
H	–1.439579000000	–1.599463000000	0.000057000000
C	–0.265646000000	0.101978000000	–0.000073000000
C	–0.156019000000	1.532570000000	–0.000074000000
N	–0.080120000000	2.680826000000	–0.000323000000
C	0.992103000000	–0.706579000000	–0.000105000000
O	1.011305000000	–1.911433000000	–0.000264000000

O	2.071656000000	0.064735000000	0.000010000000
C	3.349077000000	–0.612530000000	–0.000031000000
H	3.398169000000	–1.247400000000	0.885797000000
H	3.398321000000	–1.246989000000	–0.886146000000
C	4.420165000000	0.451599000000	0.000306000000
H	4.339939000000	1.079690000000	0.888397000000
H	4.340085000000	1.080110000000	–0.887502000000
H	5.401681000000	–0.024397000000	0.000273000000

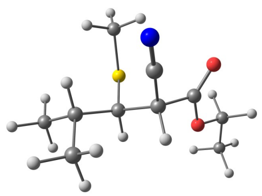

ThioRas-MeSH-*syn*-adduct

Number of imaginary frequencies: 0

Optimized energy = –994.610048781

Sum of electronic and zero-point Energies = –994.348445

Sum of electronic and thermal Enthalpies = –994.330477

Sum of electronic and thermal Free Energies = –994.393773

C	–1.333558000000	0.795689000000	–0.351381000000
O	–2.074885000000	–0.272041000000	–0.597415000000
C	–3.420780000000	–0.254934000000	–0.066100000000
H	–3.951010000000	0.590922000000	–0.505474000000
H	–3.353183000000	–0.104488000000	1.012806000000
O	–1.712084000000	1.793405000000	0.202505000000
C	2.275506000000	–0.716953000000	–0.454596000000
H	2.789053000000	0.166918000000	–0.060177000000
C	2.889579000000	–1.964042000000	0.181590000000
H	2.831128000000	–1.935739000000	1.269271000000
H	3.939409000000	–2.047065000000	–0.105554000000
H	2.371343000000	–2.863222000000	–0.166454000000
C	2.484937000000	–0.753322000000	–1.973821000000
H	2.210287000000	0.183131000000	–2.463348000000

H	1.900611000000	–1.562884000000	–2.422761000000
H	3.537520000000	–0.937467000000	–2.195385000000
C	0.778132000000	–0.601516000000	–0.116799000000
H	0.273581000000	–1.508548000000	–0.462470000000
C	0.082998000000	0.571866000000	–0.869782000000
H	0.007751000000	0.298545000000	–1.926800000000
S	0.386887000000	–0.600388000000	1.672372000000
C	1.134847000000	0.952804000000	2.244102000000
H	2.187224000000	1.010991000000	1.968382000000
H	1.057031000000	0.933970000000	3.330480000000
H	0.591069000000	1.816445000000	1.864571000000
C	0.847776000000	1.819086000000	–0.803974000000
N	1.472964000000	2.781947000000	–0.777850000000
C	–4.059289000000	–1.576395000000	–0.419236000000
H	–4.098404000000	–1.709700000000	–1.501068000000
H	–5.078251000000	–1.599903000000	–0.030452000000
H	–3.501238000000	–2.403927000000	0.020699000000

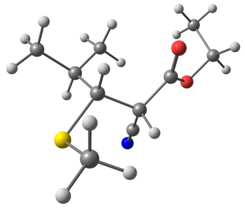

ThioRas-MeSH-*anti*-adduct

Number of imaginary frequencies: 0

Optimized energy = –994.608912269

Sum of electronic and zero-point Energies = –994.347133

Sum of electronic and thermal Enthalpies = –994.329292

Sum of electronic and thermal Free Energies = –994.392033

C	–1.232339000000	–0.683832000000	–0.510869000000
O	–2.263710000000	–0.867736000000	0.296133000000
C	–3.586265000000	–0.728360000000	–0.282640000000
H	–3.594552000000	–1.232727000000	–1.248311000000
H	–4.234513000000	–1.259490000000	0.410373000000

O	–1.300879000000	–0.455371000000	–1.689267000000
C	0.999544000000	1.578987000000	0.364456000000
H	1.312053000000	1.506618000000	1.413663000000
C	1.886024000000	2.606238000000	–0.343929000000
H	2.939577000000	2.330184000000	–0.313386000000
H	1.773055000000	3.583455000000	0.129223000000
H	1.585389000000	2.702027000000	–1.391780000000
C	–0.455514000000	2.058838000000	0.333801000000
H	–1.124141000000	1.432621000000	0.929869000000
H	–0.830065000000	2.095516000000	–0.693375000000
H	–0.512290000000	3.067476000000	0.746999000000
C	1.165982000000	0.184116000000	–0.270560000000
H	1.061058000000	0.261211000000	–1.356083000000
C	0.114815000000	–0.854992000000	0.197899000000
H	0.450529000000	–1.851048000000	–0.109603000000
S	2.850402000000	–0.438245000000	0.081081000000
C	2.992982000000	–1.722922000000	–1.191003000000
H	2.305278000000	–2.549926000000	–1.015248000000
H	4.012180000000	–2.102559000000	–1.134499000000
H	2.824712000000	–1.295445000000	–2.178797000000
C	0.000221000000	–0.878200000000	1.657102000000
N	–0.055000000000	–0.876503000000	2.804249000000
C	–3.963806000000	0.733347000000	–0.400370000000
H	–4.984557000000	0.811446000000	–0.778323000000
H	–3.299522000000	1.253806000000	–1.091321000000
H	–3.918759000000	1.219806000000	0.575308000000

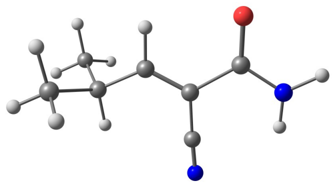

iPrCAA

Number of imaginary frequencies: 0

Optimized energy = –457.436172662

Sum of electronic and zero-point Energies = –457.272423

Sum of electronic and thermal Enthalpies = –457.260151

Sum of electronic and thermal Free Energies = –457.310340

C	–0.595415000000	–0.520040000000	–0.000628000000
C	0.599095000000	0.087391000000	–0.000812000000
C	1.864646000000	–0.734040000000	0.000758000000
O	1.815898000000	–1.953156000000	0.002272000000
C	–1.935561000000	0.134808000000	–0.001560000000
H	–1.811449000000	1.221176000000	–0.006709000000
H	–0.575776000000	–1.608963000000	0.000771000000
C	–2.704486000000	–0.287079000000	–1.261500000000
H	–2.177244000000	0.013553000000	–2.168426000000
H	–3.691065000000	0.180271000000	–1.262640000000
H	–2.839992000000	–1.371683000000	–1.282254000000
C	–2.699734000000	–0.275282000000	1.265289000000
H	–2.169147000000	0.034108000000	2.167302000000
H	–2.834984000000	–1.359639000000	1.296900000000
H	–3.686387000000	0.191923000000	1.265531000000
C	0.703414000000	1.517420000000	–0.002396000000
N	0.829628000000	2.662150000000	–0.003586000000
N	3.017136000000	–0.038772000000	0.000219000000
H	3.050982000000	0.968719000000	–0.001097000000
H	3.888763000000	–0.546928000000	0.001107000000

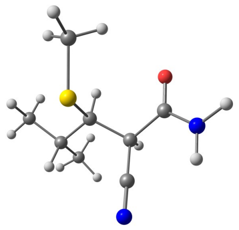

iPrCAA-MeSH-*syn*-adduct

Number of imaginary frequencies: 0

Optimized energy = –896.144591497

Sum of electronic and zero-point Energies = –895.927779

Sum of electronic and thermal Enthalpies = –895.912287

Sum of electronic and thermal Free Energies = –895.969287

C	–1.914368000000	0.037564000000	0.749256000000
O	–1.816300000000	0.893122000000	1.610910000000
C	1.871949000000	–0.704995000000	0.031855000000
H	1.847728000000	–1.125511000000	–0.980628000000
C	3.069108000000	0.240359000000	0.141062000000
H	2.990354000000	1.072080000000	–0.560828000000
H	3.995727000000	–0.296938000000	–0.068945000000
H	3.136497000000	0.647469000000	1.154909000000
C	2.022228000000	–1.854172000000	1.034070000000
H	1.294185000000	–2.651941000000	0.872173000000
H	1.914307000000	–1.488952000000	2.060597000000
H	3.015388000000	–2.297171000000	0.940234000000
C	0.565805000000	0.076511000000	0.252071000000
H	0.639401000000	0.660211000000	1.172671000000
C	–0.677938000000	–0.823126000000	0.424880000000
H	–0.535731000000	–1.469502000000	1.297351000000
S	0.250841000000	1.278548000000	–1.105692000000
C	0.331771000000	2.837404000000	–0.176399000000
H	–0.422434000000	2.839931000000	0.609073000000
H	0.128244000000	3.638157000000	–0.886009000000
H	1.326802000000	2.974675000000	0.245962000000
C	–0.856895000000	–1.693820000000	–0.737449000000
N	–0.996127000000	–2.365507000000	–1.659633000000
N	–3.047735000000	–0.232104000000	0.085255000000
H	–3.102852000000	–0.923461000000	–0.646195000000
H	–3.873600000000	0.308135000000	0.297997000000

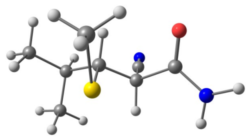

iPrCAA-MeSH-*anti*-adduct

Number of imaginary frequencies: 0

Optimized energy = –896.144131617

Sum of electronic and zero-point Energies = –895.927300

Sum of electronic and thermal Enthalpies = –895.911680

Sum of electronic and thermal Free Energies = –895.969208

C	–2.020677000000	–0.390331000000	–0.033955000000
O	–2.077473000000	–0.918388000000	–1.128991000000
C	1.605480000000	1.086394000000	–0.159146000000
H	1.285003000000	1.938281000000	–0.771072000000
C	1.878105000000	1.588794000000	1.258349000000
H	1.017239000000	2.107097000000	1.688011000000
H	2.713173000000	2.291865000000	1.250844000000
H	2.144868000000	0.757176000000	1.916932000000
C	2.871727000000	0.513535000000	–0.793983000000
H	2.679579000000	0.156983000000	–1.809284000000
H	3.257608000000	–0.320735000000	–0.202393000000
H	3.646709000000	1.280861000000	–0.844257000000
C	0.458435000000	0.065113000000	–0.222935000000
H	0.302804000000	–0.234742000000	–1.261338000000
C	–0.889727000000	0.610053000000	0.295579000000
H	–0.839729000000	0.779829000000	1.374218000000
S	0.800958000000	–1.488035000000	0.701719000000
C	0.966481000000	–2.640835000000	–0.691858000000
H	0.047798000000	–2.646216000000	–1.277383000000
H	1.135475000000	–3.628748000000	–0.265615000000
H	1.817235000000	–2.366511000000	–1.314340000000
C	–1.226485000000	1.891274000000	–0.335129000000
N	–1.487632000000	2.889078000000	–0.841859000000
N	–2.907642000000	–0.592100000000	0.951367000000
H	–2.817769000000	–0.156772000000	1.855319000000
H	–3.678667000000	–1.225527000000	0.796707000000

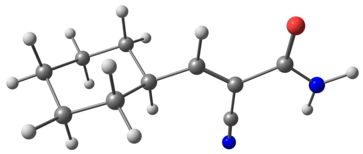

CyCAA

Number of imaginary frequencies: 0

Optimized energy = –574.153486972

Sum of electronic and zero-point Energies = –573.922838

Sum of electronic and thermal Enthalpies = –573.909037

Sum of electronic and thermal Free Energies = –573.963253

C	–3.844397000000	–0.232635000000	0.000769000000
H	–3.967532000000	–1.322331000000	0.002301000000
H	–4.846695000000	0.202942000000	0.000508000000
C	–3.084598000000	0.188250000000	1.259845000000
H	–3.034739000000	1.282515000000	1.304635000000
H	–3.614725000000	–0.144221000000	2.155723000000
C	–1.664200000000	–0.379340000000	1.264855000000
H	–1.707157000000	–1.474992000000	1.295751000000
H	–1.121069000000	–0.050152000000	2.154476000000
C	–0.896344000000	0.050738000000	–0.000614000000
H	–0.810726000000	1.143459000000	–0.002128000000
C	–1.665050000000	–0.382863000000	–1.264317000000
H	–1.708068000000	–1.478599000000	–1.292088000000
H	–1.122526000000	–0.056224000000	–2.155248000000
C	–3.085425000000	0.184774000000	–1.259962000000
H	–3.616150000000	–0.150159000000	–2.154567000000
H	–3.035584000000	1.278910000000	–1.307812000000
C	0.458217000000	–0.563518000000	–0.000106000000
H	0.510659000000	–1.651184000000	0.000534000000
C	1.635404000000	0.078185000000	–0.000245000000
C	1.698157000000	1.510492000000	–0.001030000000
N	1.790973000000	2.658522000000	–0.001658000000
C	2.923979000000	–0.705820000000	0.000394000000
O	2.911156000000	–1.926056000000	0.001718000000

N	4.055910000000	0.022635000000	–0.000058000000
H	4.060462000000	1.030720000000	–0.001534000000
H	4.941956000000	–0.459925000000	0.000186000000

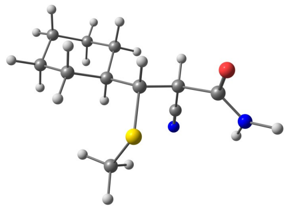

CyCAA-MeSH-*syn*-adduct

Number of imaginary frequencies: 0

Optimized energy = –1012.86140413

Sum of electronic and zero-point Energies = –1012.577451

Sum of electronic and thermal Enthalpies = –1012.560444

Sum of electronic and thermal Free Energies = –1012.621053

C	2.678488000000	–0.727053000000	–0.787533000000
O	2.703731000000	–0.598766000000	–1.998490000000
C	–1.100931000000	–0.005391000000	–0.000332000000
H	–1.079961000000	0.206233000000	1.077201000000
C	–2.071521000000	0.975877000000	–0.673350000000
H	–2.063145000000	0.780151000000	–1.753784000000
H	–1.728786000000	2.004035000000	–0.539378000000
C	–1.614292000000	–1.445398000000	–0.172112000000
H	–0.977666000000	–2.152526000000	0.366277000000
H	–1.573217000000	–1.713970000000	–1.236066000000
C	0.316855000000	0.166544000000	–0.565509000000
H	0.278881000000	0.070459000000	–1.653470000000
C	1.313818000000	–0.922899000000	–0.101342000000
H	0.959162000000	–1.896351000000	–0.455532000000
S	1.027682000000	1.845540000000	–0.319857000000
C	0.482289000000	2.299218000000	1.353440000000
H	–0.599470000000	2.421962000000	1.398238000000
H	0.954799000000	3.259745000000	1.556450000000
H	0.814527000000	1.578608000000	2.097443000000

C	1.391378000000	–1.000381000000	1.357260000000
N	1.459638000000	–1.060687000000	2.503081000000
N	3.771287000000	–0.738203000000	–0.010593000000
H	3.728952000000	–0.816002000000	0.993460000000
H	4.675412000000	–0.624729000000	–0.445219000000
C	–3.048489000000	–1.598073000000	0.342760000000
H	–3.055801000000	–1.425376000000	1.425688000000
H	–3.385242000000	–2.625368000000	0.182563000000
C	–3.999890000000	–0.609090000000	–0.328260000000
H	–5.008326000000	–0.717253000000	0.079271000000
H	–4.059707000000	–0.834080000000	–1.399946000000
C	–3.497342000000	0.821849000000	–0.143084000000
H	–3.514207000000	1.077060000000	0.923631000000
H	–4.157608000000	1.529899000000	–0.650239000000

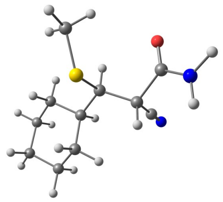

CyCAA-MeSH-anti-adduct

Number of imaginary frequencies: 0

Optimized energy = –1012.86068310

Sum of electronic and zero-point Energies = –1012.577111

Sum of electronic and thermal Enthalpies = –1012.559808

Sum of electronic and thermal Free Energies = –1012.621722

C	2.812141000000	–0.370286000000	–0.010614000000
O	3.110619000000	0.179234000000	–1.054700000000
C	–1.090775000000	–0.252493000000	–0.291813000000
H	–1.108158000000	–1.120371000000	–0.966716000000
C	–1.629516000000	–0.707287000000	1.068990000000
H	–1.577146000000	0.134121000000	1.771117000000
H	–1.010509000000	–1.510973000000	1.480527000000
C	–2.000491000000	0.815311000000	–0.909283000000

H	–1.975742000000	1.711902000000	–0.278685000000
H	–1.616276000000	1.100135000000	–1.894329000000
C	0.366774000000	0.225427000000	–0.248081000000
H	0.676599000000	0.515762000000	–1.254324000000
C	1.363556000000	–0.849442000000	0.237398000000
H	1.211857000000	–1.052572000000	1.300606000000
S	0.633754000000	1.711026000000	0.804661000000
C	1.022720000000	2.930313000000	–0.483955000000
H	1.892108000000	2.601275000000	–1.052165000000
H	1.249424000000	3.866120000000	0.025150000000
H	0.166427000000	3.075757000000	–1.141585000000
C	1.181516000000	–2.116204000000	–0.480610000000
N	1.039624000000	–3.102037000000	–1.053960000000
N	3.676095000000	–0.630094000000	0.981571000000
H	3.392266000000	–1.067165000000	1.843565000000
H	4.642645000000	–0.357835000000	0.875592000000
C	–3.444047000000	0.320983000000	–1.021476000000
H	–3.484404000000	–0.528394000000	–1.713966000000
H	–4.074258000000	1.107597000000	–1.444231000000
C	–3.977941000000	–0.116582000000	0.343480000000
H	–4.011379000000	0.752480000000	1.011781000000
H	–5.001668000000	–0.488464000000	0.250414000000
C	–3.078716000000	–1.189479000000	0.959255000000
H	–3.109623000000	–2.087887000000	0.330989000000
H	–3.448541000000	–1.478430000000	1.946276000000

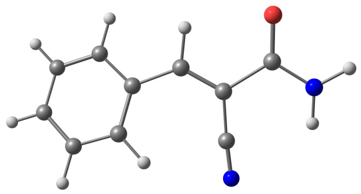

PhCAA

Number of imaginary frequencies: 0

Optimized energy = –570.537266950

Sum of electronic and zero-point Energies = –570.376516

Sum of electronic and thermal Enthalpies = –570.364081

Sum of electronic and thermal Free Energies = –570.415536

C	–0.309942000000	–0.738217000000	0.096531000000
C	–1.415591000000	0.026971000000	0.013961000000
C	–2.763135000000	–0.655104000000	0.058539000000
O	–2.846051000000	–1.864438000000	0.198934000000
H	–0.518065000000	–1.803052000000	0.167106000000
C	–1.396393000000	1.445193000000	–0.160237000000
N	–1.452238000000	2.585896000000	–0.312360000000
N	–3.835311000000	0.148830000000	–0.066068000000
H	–3.765162000000	1.146998000000	–0.187594000000
H	–4.753898000000	–0.267674000000	–0.044733000000
C	1.101596000000	–0.370238000000	0.072725000000
C	3.845953000000	0.163107000000	–0.032851000000
C	1.584068000000	0.922140000000	0.330614000000
C	2.019960000000	–1.392192000000	–0.212532000000
C	3.380830000000	–1.126056000000	–0.276904000000
C	2.946383000000	1.181186000000	0.279151000000
H	0.906334000000	1.722243000000	0.595572000000
H	1.655575000000	–2.397646000000	–0.391711000000
H	4.076712000000	–1.922798000000	–0.508685000000
H	3.309501000000	2.179929000000	0.488221000000
H	4.907882000000	0.373676000000	–0.074637000000

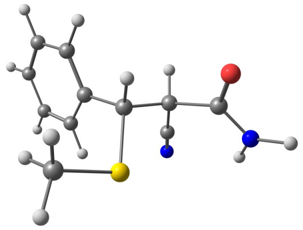

PhCAA-MeSH-*syn*-adduct

Number of imaginary frequencies: 0

Optimized energy = –1009.24455884

Sum of electronic and zero-point Energies = –1009.031451

Sum of electronic and thermal Enthalpies = –1009.015351

Sum of electronic and thermal Free Energies = –1009.075267

C	–2.622282000000	–0.433150000000	0.831600000000
O	–2.770721000000	0.196916000000	1.861965000000
C	–0.269583000000	0.407438000000	0.400243000000
H	–0.443843000000	0.905207000000	1.355952000000
C	–1.188044000000	–0.838398000000	0.424160000000
H	–0.828021000000	–1.495529000000	1.222349000000
S	–0.854335000000	1.536675000000	–0.915656000000
C	0.250196000000	2.932707000000	–0.574401000000
H	0.109308000000	3.284595000000	0.447101000000
H	–0.019391000000	3.724713000000	–1.271040000000
H	1.287807000000	2.642668000000	–0.736939000000
C	–1.122688000000	–1.598461000000	–0.824986000000
N	–1.102018000000	–2.196986000000	–1.805605000000
N	–3.624778000000	–0.812797000000	0.026863000000
H	–3.478440000000	–1.329948000000	–0.826078000000
H	–4.570180000000	–0.568402000000	0.283828000000
C	1.193146000000	0.028461000000	0.297377000000
C	3.882778000000	–0.736052000000	0.205371000000
C	1.953294000000	–0.030397000000	1.466292000000
C	1.794840000000	–0.290426000000	–0.921547000000
C	3.131488000000	–0.673174000000	–0.965689000000
C	3.291287000000	–0.412154000000	1.422409000000
H	1.495342000000	0.224911000000	2.416515000000
H	1.219572000000	–0.223780000000	–1.838217000000
H	3.588492000000	–0.916721000000	–1.917488000000
H	3.870330000000	–0.450404000000	2.337378000000
H	4.925121000000	–1.029304000000	0.167636000000

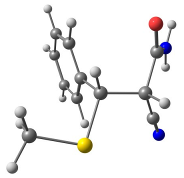

PhCAA-MeSH-anti-adduct

Number of imaginary frequencies: 0

Optimized energy = –1009.24538446

Sum of electronic and zero-point Energies = –1009.032195

Sum of electronic and thermal Enthalpies = –1009.016098

Sum of electronic and thermal Free Energies = –1009.076005

C	1.616999000000	–1.587888000000	–0.687521000000
O	1.674396000000	–1.699819000000	–1.898360000000
C	0.556678000000	0.683616000000	–0.592735000000
H	0.643734000000	0.668058000000	–1.680417000000
C	1.743459000000	–0.175003000000	–0.080669000000
H	2.672797000000	0.245454000000	–0.475523000000
S	0.819242000000	2.408579000000	–0.045471000000
C	–0.603218000000	3.167138000000	–0.874248000000
H	–0.552745000000	2.992256000000	–1.948598000000
H	–0.545985000000	4.236685000000	–0.678597000000
H	–1.534203000000	2.772507000000	–0.468590000000
C	1.844096000000	–0.175888000000	1.379344000000
N	1.926599000000	–0.209226000000	2.524928000000
N	1.420023000000	–2.608961000000	0.157851000000
H	1.318040000000	–3.538903000000	–0.222185000000
H	1.367170000000	–2.490840000000	1.157793000000
C	–0.781778000000	0.096902000000	–0.197063000000
C	–3.213938000000	–1.102476000000	0.488901000000
C	–1.302176000000	0.242895000000	1.091015000000
C	–1.496466000000	–0.644178000000	–1.139477000000
C	–2.706523000000	–1.242905000000	–0.798699000000
C	–2.509899000000	–0.356583000000	1.431748000000
H	–0.768203000000	0.839616000000	1.822091000000

H	–1.099540000000	–0.755358000000	–2.143199000000
H	–3.252052000000	–1.813844000000	–1.540573000000
H	–2.905161000000	–0.235790000000	2.433351000000
H	–4.156659000000	–1.565030000000	0.755833000000

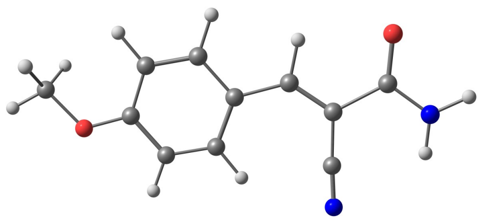

4OMeCAA

Number of imaginary frequencies: 0

Optimized energy = –685.055082349

Sum of electronic and zero-point Energies = –684.861366

Sum of electronic and thermal Enthalpies = –684.846392

Sum of electronic and thermal Free Energies = –684.903717

C	–1.158917000000	–0.707870000000	–0.000460000000
C	–2.299114000000	0.018745000000	–0.000063000000
C	–3.612311000000	–0.721168000000	–0.000224000000
O	–3.646375000000	–1.942312000000	–0.001167000000
H	–1.337632000000	–1.780537000000	–0.000736000000
C	–2.347264000000	1.444302000000	0.000778000000
N	–2.454587000000	2.592203000000	0.001610000000
N	–4.722348000000	0.042380000000	0.000456000000
H	–4.698530000000	1.049991000000	0.002559000000
H	–5.620893000000	–0.415873000000	0.001174000000
C	0.236915000000	–0.319268000000	–0.000461000000
C	2.994898000000	0.218915000000	–0.000191000000
C	0.713082000000	1.008499000000	–0.001767000000
C	1.182924000000	–1.354173000000	0.000825000000
C	2.547422000000	–1.104513000000	0.001050000000
C	2.064741000000	1.270992000000	–0.001653000000
H	0.027793000000	1.844503000000	–0.003035000000

H	0.839402000000	–2.382732000000	0.001731000000
H	3.241109000000	–1.933380000000	0.002129000000
H	2.436154000000	2.288286000000	–0.002734000000
O	4.290152000000	0.581019000000	–0.000138000000
C	5.275927000000	–0.446298000000	0.000802000000
H	5.187750000000	–1.066030000000	0.895861000000
H	6.235647000000	0.062751000000	0.000315000000
H	5.187716000000	–1.067693000000	–0.893097000000

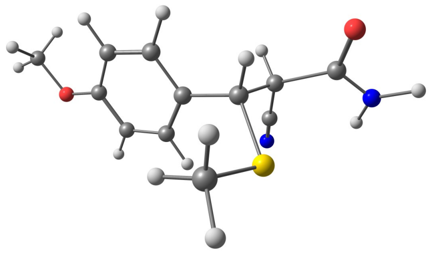

4OMeCAA-MeSH-*syn*-adduct

Number of imaginary frequencies: 0

Optimized energy = –1123.75976822

Sum of electronic and zero-point Energies = –1123.513778

Sum of electronic and thermal Enthalpies = –1123.495122

Sum of electronic and thermal Free Energies = –1123.561033

C	–3.212427000000	–0.694344000000	0.928842000000
O	–3.355471000000	–0.137172000000	2.001341000000
C	–0.988645000000	0.411311000000	0.408750000000
H	–1.146705000000	0.832745000000	1.403194000000
C	–1.775281000000	–0.922539000000	0.410761000000
H	–1.298000000000	–1.581037000000	1.143497000000
S	–1.777889000000	1.546072000000	–0.793027000000
C	–0.793534000000	3.026887000000	–0.441976000000
H	–0.894992000000	3.307477000000	0.605979000000
H	–1.187383000000	3.823847000000	–1.070455000000
H	0.253265000000	2.851737000000	–0.688200000000
C	–1.716487000000	–1.602404000000	–0.883948000000
N	–1.698574000000	–2.141033000000	–1.898780000000

N	–4.223164000000	–1.137656000000	0.167853000000
H	–5.168872000000	–1.009796000000	0.497732000000
H	–4.083291000000	–1.594281000000	–0.719992000000
C	0.492407000000	0.204042000000	0.187118000000
C	3.243658000000	–0.242146000000	–0.132485000000
C	1.038049000000	–0.011459000000	–1.084273000000
C	1.349024000000	0.202701000000	1.282548000000
C	2.718491000000	–0.019484000000	1.139141000000
C	2.393789000000	–0.233935000000	–1.244669000000
H	0.395206000000	0.011568000000	–1.957127000000
H	0.948910000000	0.378309000000	2.275914000000
H	3.351552000000	–0.009756000000	2.015681000000
H	2.821195000000	–0.397813000000	–2.226611000000
O	4.556519000000	–0.465316000000	–0.389329000000
C	5.453125000000	–0.474824000000	0.711931000000
H	5.454175000000	0.491220000000	1.223170000000
H	6.438388000000	–0.667582000000	0.295507000000
H	5.193539000000	–1.265892000000	1.420108000000

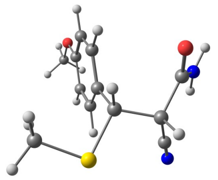

4OMeCAA-MeSH-*anti*-adduct

Number of imaginary frequencies: 0

Optimized energy = –1123.76050796

Sum of electronic and zero-point Energies = –1123.514512

Sum of electronic and thermal Enthalpies = –1123.495865

Sum of electronic and thermal Free Energies = –1123.561735

C	1.832501000000	–1.976395000000	–0.532285000000
O	2.082519000000	–2.077504000000	–1.719528000000
C	1.474633000000	0.505614000000	–0.484405000000
H	1.771034000000	0.470972000000	–1.534125000000

C	2.231487000000	–0.671239000000	0.186982000000
H	3.304202000000	–0.542161000000	0.015546000000
S	2.120005000000	2.069035000000	0.213282000000
C	1.179912000000	3.225875000000	–0.817523000000
H	1.386652000000	3.045971000000	–1.872222000000
H	1.512692000000	4.227221000000	–0.549032000000
H	0.112968000000	3.130683000000	–0.618946000000
C	2.016512000000	–0.714900000000	1.633882000000
N	1.839910000000	–0.779694000000	2.767450000000
N	1.206420000000	–2.915737000000	0.190160000000
H	1.006791000000	–2.809701000000	1.172747000000
H	0.926832000000	–3.771317000000	–0.267441000000
C	–0.026343000000	0.347165000000	–0.391057000000
C	–2.797981000000	–0.067345000000	–0.260362000000
C	–0.728870000000	–0.151618000000	–1.493537000000
C	–0.738202000000	0.643664000000	0.768132000000
C	–2.114060000000	0.440120000000	0.845421000000
C	–2.096660000000	–0.359397000000	–1.433701000000
H	–0.193189000000	–0.383726000000	–2.408197000000
H	–0.220090000000	1.056613000000	1.626707000000
H	–2.633543000000	0.686250000000	1.761351000000
H	–2.643000000000	–0.743182000000	–2.286809000000
O	–4.133871000000	–0.298996000000	–0.291067000000
C	–4.882645000000	–0.013492000000	0.881530000000
H	–4.536220000000	–0.618546000000	1.723242000000
H	–5.913271000000	–0.268745000000	0.649192000000
H	–4.817138000000	1.046806000000	1.138492000000

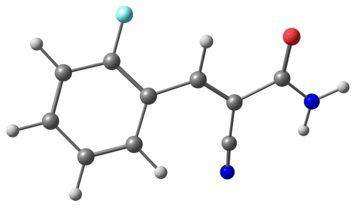

2FCAA

Number of imaginary frequencies: 0

Optimized energy = –669.777568911

Sum of electronic and zero-point Energies = –669.624973

Sum of electronic and thermal Enthalpies = –669.611749

Sum of electronic and thermal Free Energies = –669.664853

C	–0.222752000000	1.967766000000	–0.897299000000
C	0.306641000000	2.275289000000	1.783443000000
C	0.090512000000	0.827363000000	–0.151958000000
C	–0.298180000000	3.232699000000	–0.350251000000
C	–0.031971000000	3.381819000000	1.006252000000
C	0.366679000000	1.013692000000	1.211420000000
H	–0.558286000000	4.072398000000	–0.981682000000
H	–0.081784000000	4.366585000000	1.453867000000
H	0.661922000000	0.168489000000	1.818725000000
H	0.532626000000	2.398955000000	2.834615000000
F	–0.477622000000	1.822067000000	–2.209582000000
C	0.153549000000	–0.454304000000	–0.843017000000
H	0.334383000000	–0.422354000000	–1.913461000000
C	–0.003813000000	–1.692166000000	–0.340814000000
C	–0.345064000000	–1.956376000000	1.022559000000
N	–0.633594000000	–2.232729000000	2.102896000000
C	0.131361000000	–2.870213000000	–1.279023000000
O	0.399097000000	–2.698309000000	–2.456398000000
N	–0.062153000000	–4.083406000000	–0.731329000000
H	–0.280549000000	–4.220111000000	0.243299000000
H	0.019002000000	–4.897154000000	–1.322263000000

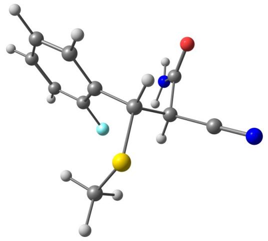

2FCAA-MeSH-*syn*-adduct

Number of imaginary frequencies: 0

Optimized energy = –1108.48594982

Sum of electronic and zero-point Energies = –1108.280278

Sum of electronic and thermal Enthalpies = –1108.263667

Sum of electronic and thermal Free Energies = –1108.323659

C	1.160279000000	–1.894293000000	0.020090000000
O	1.285607000000	–2.383737000000	–1.087343000000
C	0.591938000000	0.468223000000	–0.625365000000
H	0.851093000000	0.252789000000	–1.663777000000
C	1.522751000000	–0.411415000000	0.251476000000
H	1.437981000000	–0.141154000000	1.303787000000
S	0.965150000000	2.254599000000	–0.477178000000
C	0.300124000000	2.651030000000	1.162013000000
H	–0.777861000000	2.495875000000	1.191474000000
H	0.511355000000	3.708924000000	1.312911000000
H	0.791913000000	2.076328000000	1.944954000000
C	2.917867000000	–0.216066000000	–0.146204000000
N	4.011903000000	–0.075242000000	–0.465901000000
N	0.688665000000	–2.543730000000	1.092997000000
H	0.577799000000	–2.094108000000	1.988260000000
H	0.398129000000	–3.506181000000	0.995936000000
C	–0.878217000000	0.159643000000	–0.462275000000
C	–3.637916000000	–0.360529000000	–0.249861000000
C	–1.498320000000	–0.069961000000	0.760542000000
C	–1.698805000000	0.120675000000	–1.594690000000
C	–3.059962000000	–0.133524000000	–1.496818000000
C	–2.850090000000	–0.330951000000	0.893590000000
H	–1.249620000000	0.294539000000	–2.566443000000
H	–3.668107000000	–0.159328000000	–2.392056000000
H	–3.259989000000	–0.503798000000	1.880596000000
H	–4.698248000000	–0.562468000000	–0.165020000000
F	–0.751508000000	–0.047699000000	1.893145000000

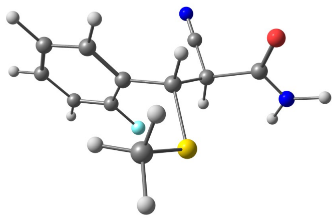

2FCAA-MeSH-anti-adduct

Number of imaginary frequencies: 0

Optimized energy = –1108.48592038

Sum of electronic and zero-point Energies = –1108.280693

Sum of electronic and thermal Enthalpies = –1108.263707

Sum of electronic and thermal Free Energies = –1108.325814

C	2.792697000000	–0.456393000000	–0.198152000000
O	3.061874000000	–0.254345000000	–1.366758000000
C	0.405191000000	0.351976000000	–0.340736000000
H	0.671187000000	0.478615000000	–1.392416000000
C	1.334019000000	–0.747125000000	0.223708000000
H	1.241886000000	–0.804037000000	1.309954000000
S	0.836393000000	1.918954000000	0.513063000000
C	–0.051184000000	3.063673000000	–0.576361000000
H	0.327328000000	2.989127000000	–1.595408000000
H	0.135432000000	4.065531000000	–0.192928000000
H	–1.121546000000	2.861210000000	–0.551161000000
C	0.952472000000	–2.051411000000	–0.323885000000
N	0.646071000000	–3.066692000000	–0.765275000000
N	3.684681000000	–0.456882000000	0.801752000000
H	4.655931000000	–0.275044000000	0.592771000000
H	3.422988000000	–0.623614000000	1.760287000000
C	–1.062876000000	0.030968000000	–0.249472000000
C	–3.815095000000	–0.515520000000	–0.093874000000
C	–1.875095000000	0.100119000000	–1.382448000000
C	–1.680910000000	–0.316393000000	0.948630000000
C	–3.029425000000	–0.592971000000	1.052855000000
C	–3.238861000000	–0.168435000000	–1.310934000000

H	–1.424151000000	0.368825000000	–2.331654000000
H	–3.443930000000	–0.861804000000	2.016274000000
H	–3.847184000000	–0.106936000000	–2.204320000000
H	–4.874966000000	–0.728014000000	–0.030349000000
F	–0.924570000000	–0.395364000000	2.065852000000

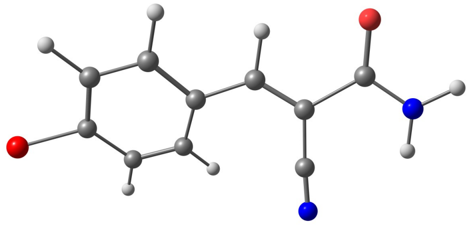

4BrCAA

Number of imaginary frequencies: 0

Optimized energy = –3144.10909139

Sum of electronic and zero-point Energies = –3143.958575

Sum of electronic and thermal Enthalpies = –3143.944710

Sum of electronic and thermal Free Energies = –3144.000604

H	0.050228000000	–2.545630000000	–0.357035000000
C	–0.365234000000	–1.560420000000	–0.179256000000
C	–1.435402000000	0.961821000000	0.305737000000
C	0.495784000000	–0.484837000000	0.082280000000
C	–1.740660000000	–1.386615000000	–0.224181000000
C	–2.260316000000	–0.121057000000	0.018153000000
C	–0.061492000000	0.777286000000	0.335297000000
H	–2.395495000000	–2.221380000000	–0.436122000000
H	0.565069000000	1.623590000000	0.580906000000
H	–1.860794000000	1.935468000000	0.509983000000
Br	–4.139786000000	0.134752000000	–0.025383000000
C	1.926579000000	–0.769532000000	0.091991000000
H	2.196298000000	–1.820833000000	0.155002000000
C	2.984272000000	0.059474000000	0.007410000000
C	2.880704000000	1.475406000000	–0.155157000000
N	2.864451000000	2.618486000000	–0.297937000000

C	4.369969000000	–0.544510000000	0.040923000000
O	4.521258000000	–1.749024000000	0.161313000000
N	5.393000000000	0.322257000000	–0.069599000000
H	5.265199000000	1.317160000000	–0.168959000000
H	6.334541000000	–0.039785000000	–0.052282000000

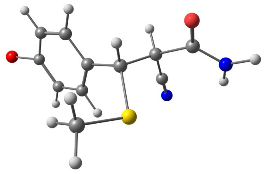

4BrCAA-MeSH-*syn*-adduct

Number of imaginary frequencies: 0

Optimized energy = –3582.81706022

Sum of electronic and zero-point Energies = –3582.614261

Sum of electronic and thermal Enthalpies = –3582.596662

Sum of electronic and thermal Free Energies = –3582.661539

C	–3.834872000000	–0.740023000000	0.861670000000
O	–4.053040000000	–0.120721000000	1.885909000000
C	–1.644216000000	0.445329000000	0.392318000000
H	–1.877271000000	0.906697000000	1.353477000000
C	–2.365503000000	–0.923506000000	0.418410000000
H	–1.895506000000	–1.529601000000	1.199925000000
S	–2.404564000000	1.481625000000	–0.909172000000
C	–1.537259000000	3.032132000000	–0.548656000000
H	–1.734412000000	3.345303000000	0.476050000000
H	–1.928639000000	3.778546000000	–1.237760000000
H	–0.466319000000	2.914068000000	–0.711212000000
C	–2.214663000000	–1.650777000000	–0.842718000000
N	–2.121138000000	–2.225856000000	–1.833039000000
N	–4.785075000000	–1.292440000000	0.094926000000
H	–4.581815000000	–1.802445000000	–0.750599000000
H	–5.749429000000	–1.202169000000	0.380361000000
C	–0.143659000000	0.290377000000	0.267430000000

C	2.608437000000	–0.057467000000	0.132087000000
C	0.480100000000	0.059606000000	–0.959514000000
C	0.636652000000	0.353743000000	1.421496000000
C	2.015789000000	0.179891000000	1.364375000000
C	1.856609000000	–0.118693000000	–1.034734000000
H	0.166747000000	0.542304000000	2.380902000000
H	2.615212000000	0.234154000000	2.263731000000
H	2.335722000000	–0.296265000000	–1.988821000000
H	–0.109447000000	0.032980000000	–1.868829000000
Br	4.494900000000	–0.293126000000	0.037030000000

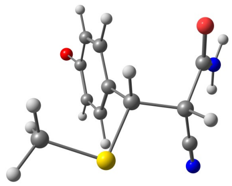

4BrCAA-MeSH-anti-adduct

Number of imaginary frequencies: 0

Optimized energy = –3582.81780496

Sum of electronic and zero-point Energies = –3582.614882

Sum of electronic and thermal Enthalpies = –3582.597346

Sum of electronic and thermal Free Energies = –3582.661746

C	2.258379000000	–2.029066000000	–0.613326000000
O	2.409706000000	–2.110435000000	–1.818412000000
C	2.044265000000	0.469320000000	–0.514102000000
H	2.277553000000	0.434641000000	–1.579386000000
C	2.768338000000	–0.757971000000	0.098981000000
H	3.834843000000	–0.683459000000	–0.133113000000
S	2.802724000000	1.986199000000	0.166798000000
C	1.876796000000	3.202655000000	–0.807456000000
H	2.020492000000	3.021789000000	–1.872316000000
H	2.278123000000	4.181169000000	–0.549058000000
H	0.817388000000	3.166749000000	–0.555666000000
C	2.634618000000	–0.811910000000	1.555286000000

N	2.518107000000	–0.883699000000	2.696099000000
N	1.651674000000	–2.958350000000	0.137332000000
H	1.306554000000	–3.793670000000	–0.313130000000
H	1.543032000000	–2.872563000000	1.136042000000
C	0.543170000000	0.386899000000	–0.335117000000
C	–2.206442000000	0.119740000000	–0.048843000000
C	–0.076599000000	0.708835000000	0.873934000000
C	–0.240672000000	–0.058684000000	–1.399606000000
C	–1.618810000000	–0.196945000000	–1.265260000000
C	–1.451528000000	0.575075000000	1.025425000000
H	0.514431000000	1.083168000000	1.702045000000
H	–2.220730000000	–0.542118000000	–2.095744000000
H	–1.927797000000	0.827564000000	1.963844000000
H	0.228461000000	–0.306326000000	–2.345592000000
Br	–4.090604000000	–0.061885000000	0.150488000000

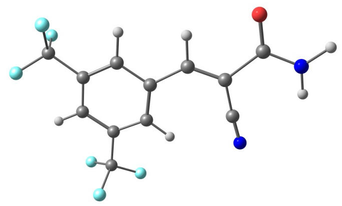

35CF$_3$CAA

Number of imaginary frequencies: 0

Optimized energy = –1244.63697859

Sum of electronic and zero-point Energies = –1244.466714

Sum of electronic and thermal Enthalpies = –1244.446998

Sum of electronic and thermal Free Energies = –1244.519008

C	1.940517000000	–0.985510000000	–0.040431000000
C	3.119042000000	–0.340393000000	–0.001301000000
C	4.383803000000	–1.176476000000	–0.024141000000
O	4.320200000000	–2.391840000000	–0.088114000000
H	2.037542000000	–2.066899000000	–0.085858000000
C	3.265560000000	1.079517000000	0.065754000000
N	3.445958000000	2.215699000000	0.120740000000

N 5.539942000000 –0.493236000000 0.030941000000
H 5.586483000000 0.512266000000 0.086177000000
H 6.405807000000 –1.011306000000 0.019270000000
C 0.566287000000 –0.485394000000 –0.027082000000
C –2.140171000000 0.275655000000 0.000392000000
C 0.208380000000 0.869600000000 –0.005487000000
C –0.450264000000 –1.447140000000 –0.038737000000
C –1.784350000000 –1.064737000000 –0.024907000000
C –1.128912000000 1.231013000000 0.009071000000
H 0.957956000000 1.647875000000 0.000842000000
H –0.191602000000 –2.499588000000 –0.061624000000
H –3.182021000000 0.572471000000 0.011699000000
C –2.853842000000 –2.120381000000 0.018848000000
C –1.507083000000 2.686306000000 –0.017791000000
F –3.118273000000 –2.502778000000 1.279910000000
F –2.495204000000 –3.222980000000 –0.651294000000
F –4.007233000000 –1.688498000000 –0.505295000000
F –2.600226000000 2.929297000000 0.718132000000
F –1.783115000000 3.100900000000 –1.266200000000
F –0.528266000000 3.471871000000 0.445582000000

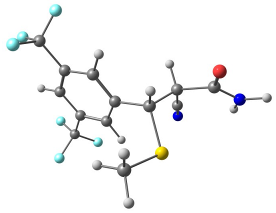

35CF$_3$CAA-MeSH-*syn*-adduct

Number of imaginary frequencies: 0

Optimized energy = –1683.34784900

Sum of electronic and zero-point Energies = –1683.125364

Sum of electronic and thermal Enthalpies = –1683.101937

Sum of electronic and thermal Free Energies = –1683.181657

C –3.966581000000 –1.020236000000 –0.751884000000
O –4.101307000000 –2.113487000000 –0.236357000000

C	–1.768813000000	–0.553009000000	0.416325000000
H	–1.914081000000	–1.576391000000	0.766258000000
C	–2.532384000000	–0.469380000000	–0.926945000000
H	–2.041502000000	–1.147423000000	–1.632917000000
S	–2.571112000000	0.562097000000	1.620934000000
C	–1.642632000000	0.067485000000	3.097674000000
H	–1.771509000000	–0.997867000000	3.285152000000
H	–2.055686000000	0.634848000000	3.929874000000
H	–0.586478000000	0.310305000000	2.984080000000
C	–2.474819000000	0.873282000000	–1.506815000000
N	–2.443508000000	1.928104000000	–1.961127000000
N	–4.976544000000	–0.261796000000	–1.198242000000
H	–4.840152000000	0.641695000000	–1.624346000000
H	–5.918916000000	–0.615351000000	–1.116479000000
C	–0.288005000000	–0.295659000000	0.230508000000
C	2.457195000000	0.110963000000	–0.201954000000
C	0.576233000000	–1.379349000000	0.103846000000
C	0.230386000000	0.996519000000	0.148311000000
C	1.587316000000	1.187357000000	–0.074277000000
C	1.934946000000	–1.170506000000	–0.111132000000
H	0.187993000000	–2.390616000000	0.166720000000
H	–0.427025000000	1.850926000000	0.264781000000
H	3.514312000000	0.268953000000	–0.373288000000
C	2.129394000000	2.587752000000	–0.119118000000
C	2.844231000000	–2.363317000000	–0.195838000000
F	3.101134000000	–2.876534000000	1.020601000000
F	2.302378000000	–3.353776000000	–0.920218000000
F	4.025700000000	–2.064871000000	–0.748918000000
F	3.258695000000	2.669837000000	–0.834580000000
F	1.253266000000	3.448907000000	–0.654651000000
F	2.416920000000	3.047514000000	1.112151000000

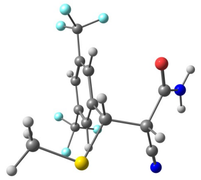

35CF₃CAA-MeSH-*anti*-adduct

Number of imaginary frequencies: 0

Optimized energy = –1683.34869379

Sum of electronic and zero-point Energies = –1683.126229

Sum of electronic and thermal Enthalpies = –1683.102773

Sum of electronic and thermal Free Energies = –1683.183111

C	–2.123554000000	–1.948898000000	–1.372835000000
O	–1.980790000000	–3.012520000000	–0.798355000000
C	–2.041522000000	–0.579711000000	0.734430000000
H	–2.075448000000	–1.544599000000	1.241742000000
C	–2.798037000000	–0.791727000000	–0.601703000000
H	–3.814431000000	–1.127284000000	–0.374061000000
S	–2.973392000000	0.617914000000	1.748708000000
C	–1.973326000000	0.517123000000	3.258028000000
H	–1.942319000000	–0.509425000000	3.621631000000
H	–2.462129000000	1.148882000000	3.997729000000
H	–0.964957000000	0.887806000000	3.076337000000
C	–2.899990000000	0.445657000000	–1.376440000000
N	–2.959696000000	1.405403000000	–2.005225000000
N	–1.718680000000	–1.704822000000	–2.625528000000
H	–1.857114000000	–0.818454000000	–3.085985000000
H	–1.287694000000	–2.454113000000	–3.147913000000
C	–0.594361000000	–0.199460000000	0.496226000000
C	2.093857000000	0.423460000000	–0.021602000000
C	0.384598000000	–1.188561000000	0.555758000000
C	–0.219482000000	1.107953000000	0.186453000000
C	1.112120000000	1.406138000000	–0.069210000000
C	1.714473000000	–0.871806000000	0.297852000000
H	0.106736000000	–2.206768000000	0.805620000000

H	–0.965282000000	1.894697000000	0.169200000000
H	3.131793000000	0.667060000000	–0.211192000000
C	2.743193000000	–1.966271000000	0.321903000000
C	1.493111000000	2.809330000000	–0.447937000000
F	2.478535000000	–2.884095000000	1.262076000000
F	2.793640000000	–2.622490000000	–0.851453000000
F	3.975955000000	–1.498522000000	0.555785000000
F	1.430433000000	2.996898000000	–1.778708000000
F	2.744997000000	3.109550000000	–0.076247000000
F	0.682394000000	3.720024000000	0.106784000000

3. Raman Shift

Table S1. Raman shifts of the peaks observed for thia-Michael reaction of 2-cyanoacrylamides with 2-mercaptoethanol in DMSO/PBS (Figure 3 and S3).

	2-cyanoacrylamide	thiol adduct
ThioRas	2236 cm^{-1}	2254 cm^{-1}
iPrCAA	2231 cm^{-1}	2247 cm^{-1}
CyCAA	2227 cm^{-1}	2246 cm^{-1}
PhCAA	2223 cm^{-1}	2248 cm^{-1}
4OMeCAA	2219 cm^{-1}	2248 cm^{-1}
2FCAA	2224 cm^{-1}	2248 cm^{-1}
4BrCAA	2223 cm^{-1}	2248 cm^{-1}
35CF_3CAA	2220 cm^{-1}	2249 cm^{-1}

4. Experimental Procedures

Turbidity assay

Nephelometric turbidity units (NTUs) were measured using the TN400 digital turbidimeter (APERA Instruments, Japan) with an infrared radiation diode (wavelength: 850 nm). Compounds were dissolved in water, mixed thoroughly, and subjected to four repeated measurements. The averaged NTU values were recorded.

Cell culture

HeLa cells were cultured in Dulbecco's modified Eagle's medium (043-30085, Wako) supplemented with 10 % fetal bovine serum, 5×10^4 U/L penicillin G, and 50 mg/L streptomycin sulfate (15070-063, Gibco), maintained at 37 °C in a 5 % CO_2 atmosphere.

General information for chemical synthesis

Proton nuclear magnetic resonance (^{1}H NMR) spectra were recorded with DMSO (δ_H 2.50) as an internal standard. Coupling constants (*J*) are reported in hertz (Hz). Abbreviations of multiplicity are as follows: s, singlet; d, doublet; t, triplet; sept, septet; m, multiplet; br, broad. Data are presented as follows: chemical shift, multiplicity, coupling constants and integration. Carbon nuclear magnetic resonance (^{13}C NMR) spectra were recorded with DMSO-*d*6 (δ_C 39.52) as an internal standard. Infrared (IR) spectra were recorded on an FT-IR spectrophotometer and absorbance bands are reported in wavenumber (cm^{-1}). Analytical thin layer chromatography (TLC) was carried out with 0.25 mm silica gel plates. Visualization was accomplished with ultraviolet light and anisaldehyde or phosphomolybdic acid stain, followed by heating. Reagents and solvents were purified by standard means or used as received unless otherwise noted. All reactions were conducted under an argon atmosphere unless otherwise noted.

Typical procedure for the synthesis of CAAs

A mixture of aldehyde, 2-cyanoacrylate, 30% aqueous NH_3, and AcOH in toluene (0.4 M) was refluxed. Water produced by the reaction was removed by azeotropic distillation using a Dean–Stark apparatus. The mixture was successively washed with saturated aqueous NH_4Cl (2 × 50 mL), saturated aqueous $NaHCO_3$ (3 × 50 mL), and brine (3 × 50 mL), and dried over anhydrous Na_2SO_4. Filtration and evaporation in vacuo furnished the crude product, which was purified to give CAAs.

Scheme S1. Synthesis of 3-substituted (*E*)-2-cyanoacrylamides.

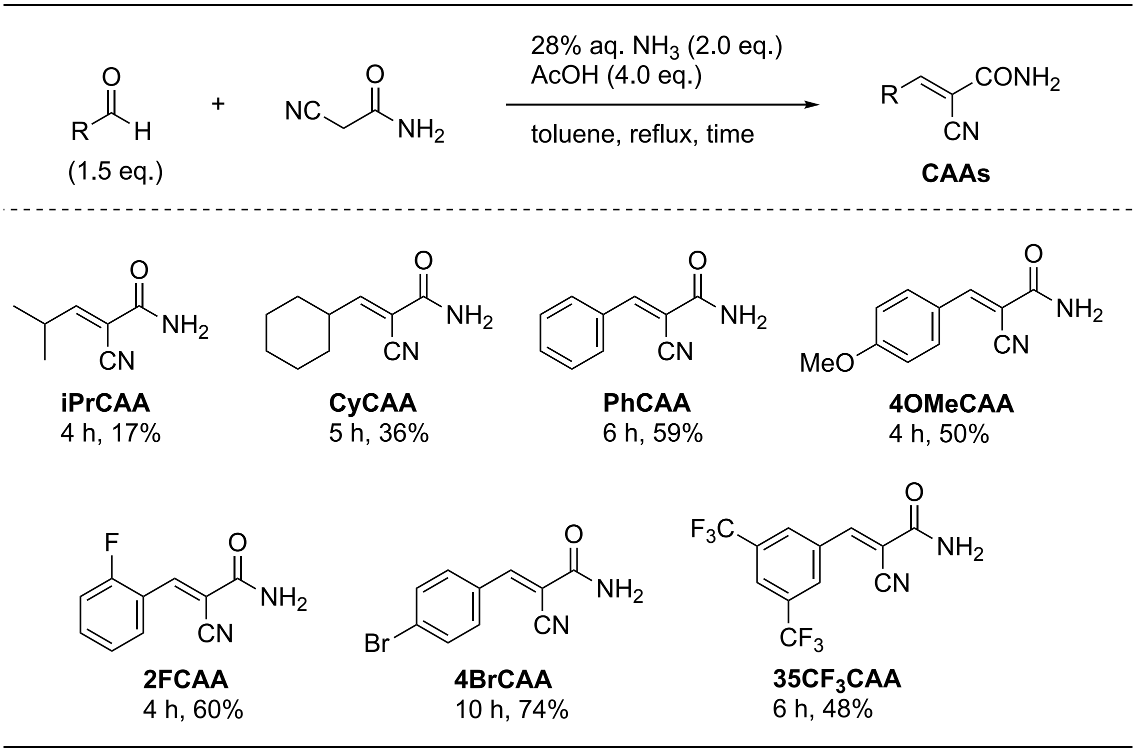

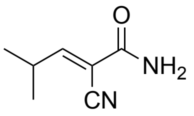

(*E*)-2-cyano-4-methylpent-2-enamide (iPrCAA)[2]: The reaction was performed according to the typical procedure employing aldehyde (2.58 g, 35.8 mmol). iPrCAA (573 mg, 17%) was obtained from the crude product after recrystallization. m.p. 79.3−85.1 °C (colorless needles from *n*-hexane/CH_3Cl); ^{1}H NMR (597 MHz, DMSO-d_6) δ = 7.79 (brs, 1H), 7.58 (brs, 1H), 7.29 (d, *J*=10.1, 1H), 2.77 (dsept, *J*=10.1, 6.7, 1H), 1.08 (d, *J*=6.7, 6H); ^{13}C NMR (150 MHz, DMSO-d_6) δ = 163.5 (CH), 162.4 (C), 114.9 (C), 110.4 (C), 31.4 (CH), 21.3 (CH_3); IR (ATR): $\tilde{n}$ = 3378, 3195, 2232, 1661, 1653, 1635, 1417, 1199, 1121, 951 cm^{-1}; HRMS (EI): *m*/*z* calcd for $C_7H_{10}N_2O$ 138.0793 [M]$^+$; found 138.0791.

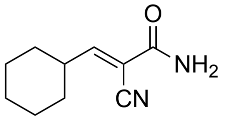

(*E*)-2-cyano-3-cyclohexylacrylamide (CyCAA)[3]: The reaction was performed according to the typical procedure employing aldehyde (4.03 g, 35.9 mmol). CyCAA (1.53 g, 36%) was obtained from the crude product after recrystallization. m.p. 126.2−127.9 °C (colorless needles from AcOEt/$CHCl_3$); ^{1}H NMR (600 MHz, DMSO-d_6) δ = 7.79 (brs, 1H), 7.58 (brs, 1H), 7.30 (d, *J*=10.0, 1H), 2.51 (m, 1H), 1.95–1.47 (m, 5H), 1.48–1.04 (m, 5H); ^{13}C NMR (151 MHz, DMSO-d_6) δ = 162.3 (C), 161.7 (CH), 114.8 (C), 110.6 (C), 40.6 (CH), 30.7 (CH_2), 25.0 (CH_2), 24.5 (CH_2); IR (ATR): $\tilde{n}$ = 3405, 2928, 2227, 1700, 1688, 1609, 1387, 964, 948 cm^{-1}; HRMS (EI): *m*/*z* calcd for $C_{10}H_{14}N_2O$ 178.1106 [M]$^+$; found 178.1115.

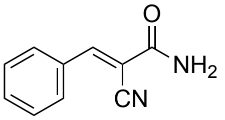

(*E*)-2-cyano-3-phenylacrylamide (PhCAA)[3]: The reaction was performed according to the typical procedure employing aldehyde (3.79 g, 35.7 mmol). PhCAA (2.42 g, 59%) was obtained as white powder from the reaction mixture after filtration. m.p. 120.2−122.2 °C; ^{1}H NMR (600 MHz, DMSO-d_6) δ = 8.19 (s, 1H), 7.94–7.93 (m, 3H), 7.78 (brs, 1H), 7.62–7.53 (m, 3H); ^{13}C NMR (151 MHz, DMSO-d_6) δ = 162.8 (C), 150.7 (CH), 132.5 (CH), 132.1 (C), 130.1 (CH), 129.4 (CH), 116.6 (C), 106.9 (C); IR (ATR): $\tilde{n}$ =3403, 3161, 2219, 1700, 1684, 1596, 1363 cm^{-1}; HRMS (EI): *m*/*z* calcd for $C_{10}H_8N_2O$ 172.0637 [M]$^+$; found 172.0645.

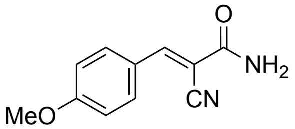

(*E*)-2-cyano-3-(4-methoxyphenyl)acrylamide (4OMeCAA)[3]: The reaction was performed according to the typical procedure employing aldehyde (4.86 g, 35.7 mmol). 4OMeCAA (2.42 g, 50%) was obtained as colorless needles from the reaction mixture after filtration. m.p. 209.8−211.9 °C; ^{1}H NMR (600 MHz, DMSO-d_6) δ = 8.11 (s, 1H), 7.96 (d, *J*=8.9, 2H), 7.79 (brs, 1H), 7.66 (brs, 1H), 7.13 (d, *J*=8.9, 2H), 3.85 (d, *J*=1.1, 4H); ^{13}C NMR (149 MHz, DMSO-d_6) δ = 163.1 (C), 162.6 (C), 150.1 (CH), 132.4 (CH), 124.4 (C), 117.0 (C), 114.8 (CH), 102.9 (C), 55.6 (CH_3); IR (ATR): $\tilde{n}$ = 3441, 3165, 2209, 1700, 1507, 1363, 1260, 1176, 1024 cm^{-1}; HRMS (EI): *m*/*z* calcd for $C_{11}H_{10}N_2O_2$ 202.0742 [M]$^+$; found 202.0742.

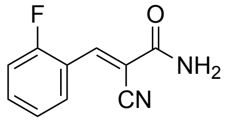

(*E*)-2-cyano-3-(2-fluorophenyl)acrylamide (2FCAA)[3]: The reaction was performed according to the typical procedure employing aldehyde (1.09 g, 5.9 mmol). 4BrCAA (718 mg, 74%) was obtained as white powder from the reaction mixture after filtration. m.p. 218.2−219.9 °C; ^{1}H NMR (600 MHz, DMSO-d_6) δ = 8.26 (s, 1H), 8.09 (td, *J*=7.6, 1.3, 1H), 8.05 (brs, 1H), 7.86 (brs, 1H), 7.65 (m, 2H), 7.45–7.38 (m, 2H); ^{13}C NMR with ^{1}H/^{19}F decoupling (151 MHz, DMSO-d_6) δ = 162.2 (C), 160.5 (C), 142.4 (CH), 134.6 (CH), 129.1 (CH), 125.3 (CH), 120.2 (C), 116.4 (CH), 115.9 (C), 109.9 (C); IR (ATR): $\tilde{n}$ = 3398, 2228, 1653, 1616, 1478, 1456, 1365, 1233, 1109, 923 cm^{-1}; HRMS (EI): *m*/*z* calcd for $C_{10}H_7FN_2O$ 190.0542 [M]$^+$; found 190.0539.

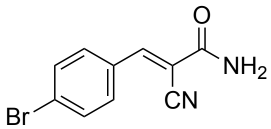

(*E*)-3-(4-bromophenyl)-2-cyanoacrylamide (4BrCAA)[3]: The reaction was performed according to the typical procedure employing aldehyde (1.09 g, 5.9 mmol). 4BrCAA (718 mg, 74%) was obtained as white powder from the reaction mixture after filtration. m.p. 218.2−219.9 °C; ^{1}H NMR (600 MHz, DMSO-d_6) δ = 8.16 (s, 1H), 7.94 (brs, 1H), 7.89–7.83 (m, 2H), 7.82–7.76 (m, 3H); ^{13}C NMR (151 MHz, DMSO-d_6) δ = 162.7 (C), 149.5 (CH), 132.5 (CH), 131.9 (CH), 131.3 (C), 126.1 (C), 116.4 (C), 107.5 (C); IR (ATR): $\tilde{n}$ = 3437, 3141, 2214, 1695, 1599, 1577, 1485, 1373, 1070, 1003, 955 cm^{-1}; HRMS (EI): *m*/*z* calcd for $C_{10}H_7BrN_2O$ 249.9742 [M]$^+$; found 249.9732.

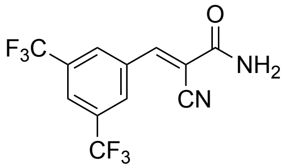

(*E*)-3-(3,5-bis(trifluoromethyl)phenyl)-2-cyanoacrylamide (35CF$_3$CAA)[4]: The reaction was performed according to the typical procedure employing aldehyde (864 mg, 3.57 mmol). 35CF$_3$CAA (351 mg, 48%) was obtained from the crude product after recrystallization. m.p. 173.2−174.7 °C (white needles from AcOEt/ Et$_2$O); ^{1}H NMR (597 MHz, DMSO-d_6) δ = 8.56 (s, 2H), 8.33 (s, 1H), 8.01 (brs, 1H), 7.93 (brs, 1H); ^{13}C NMR with ^{1}H/^{19}F decoupling (151 MHz, DMSO-d_6) δ = 161.7 (C), 147.6 (CH), 134.6 (C), 131.0 (C), 130.1 (CH), 124.9 (CH), 122.9 (C), 115.7 (C), 110.8 (C); IR (ATR): $\tilde{n}$ = 3423, 3374, 2925, 2225, 1688, 1596, 1381, 1281, 1173, 1126, 937 cm^{-1}; HRMS (EI): *m*/*z* calcd for $C_{12}H_6F_6N_2O$ 308.0384 [M]$^+$; found 308.0380.

5. Copies of ^{1}H and ^{13}C NMR Spectra

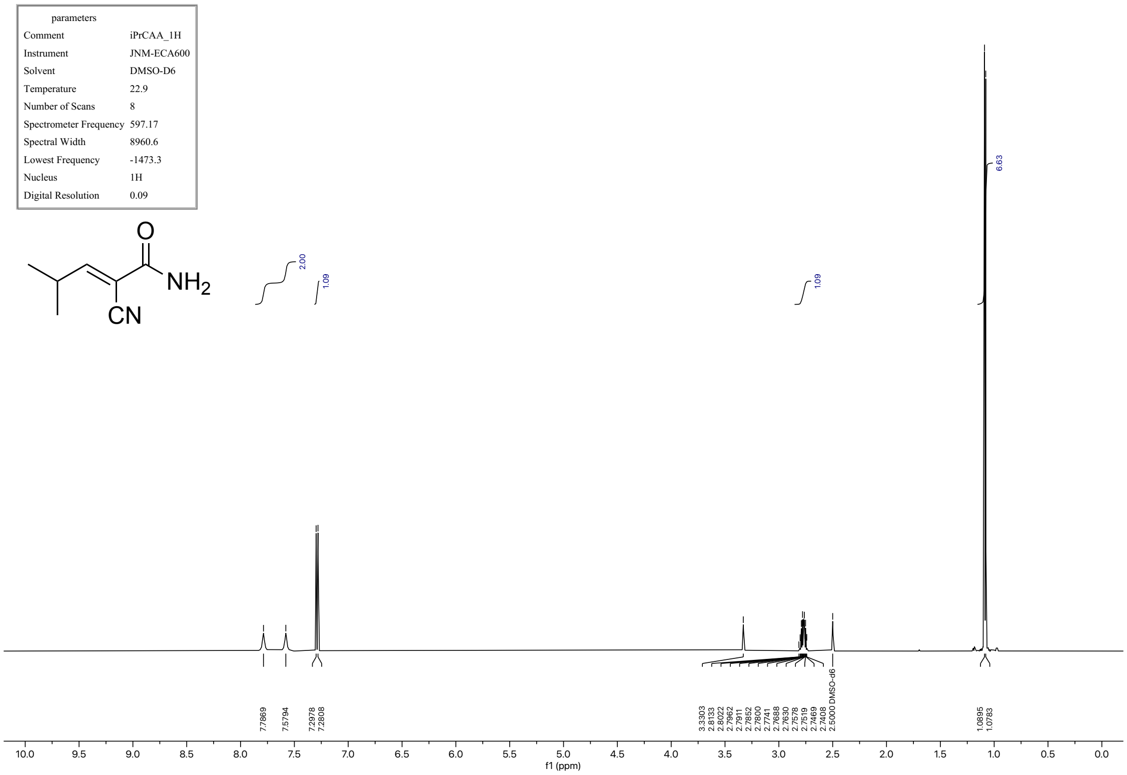

Figure S11. ^{1}H-NMR spectrum of iPrCAA.

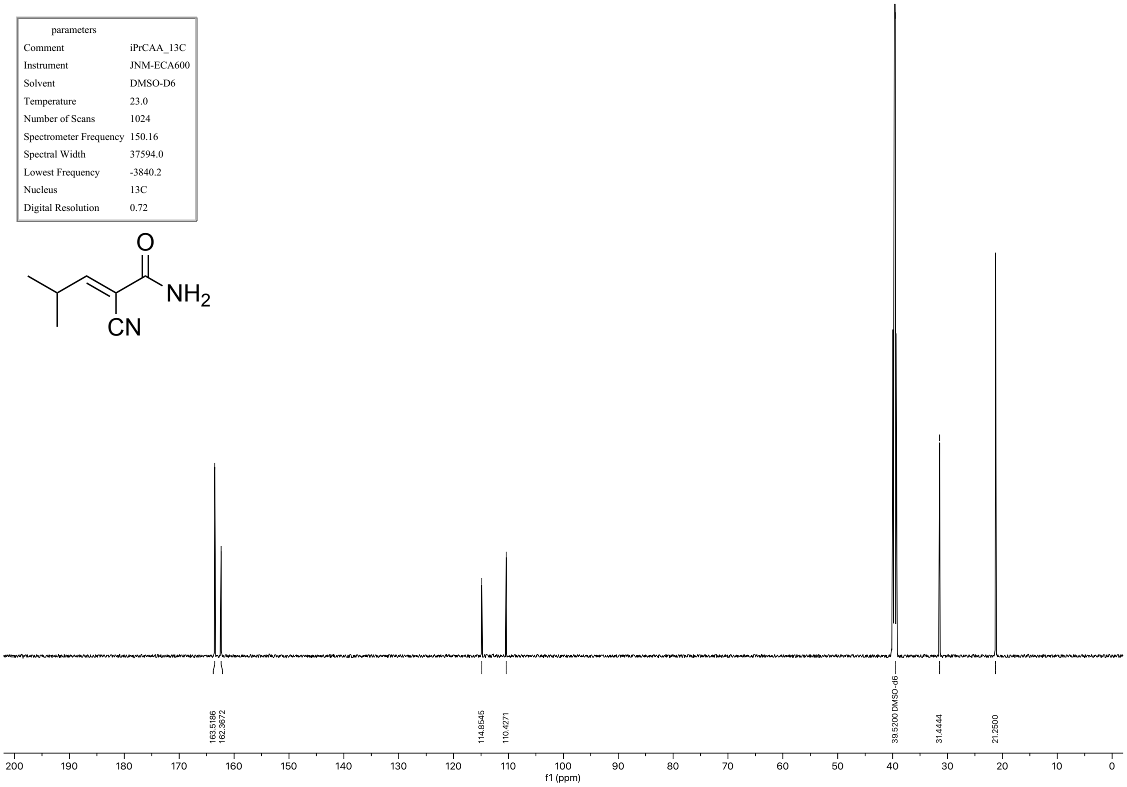

Figure S12. ^{13}C-NMR spectrum of iPrCAA.

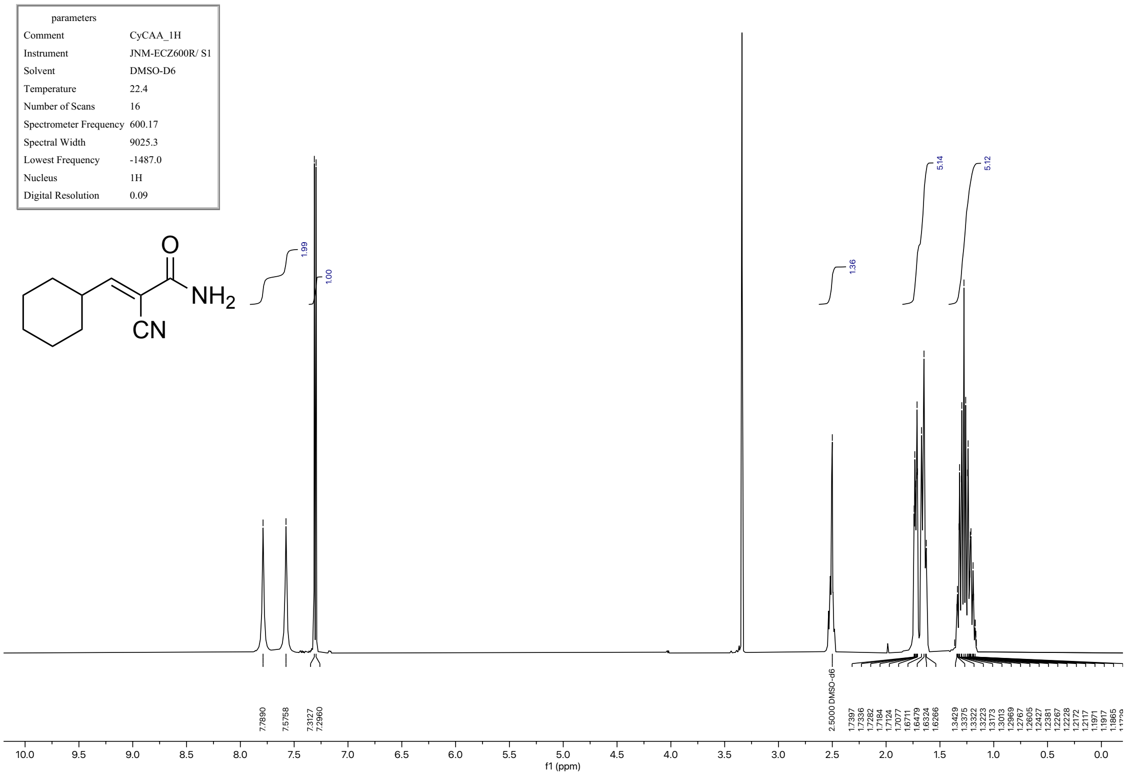

Figure S13. ^{1}H-NMR spectrum of CyCAA.

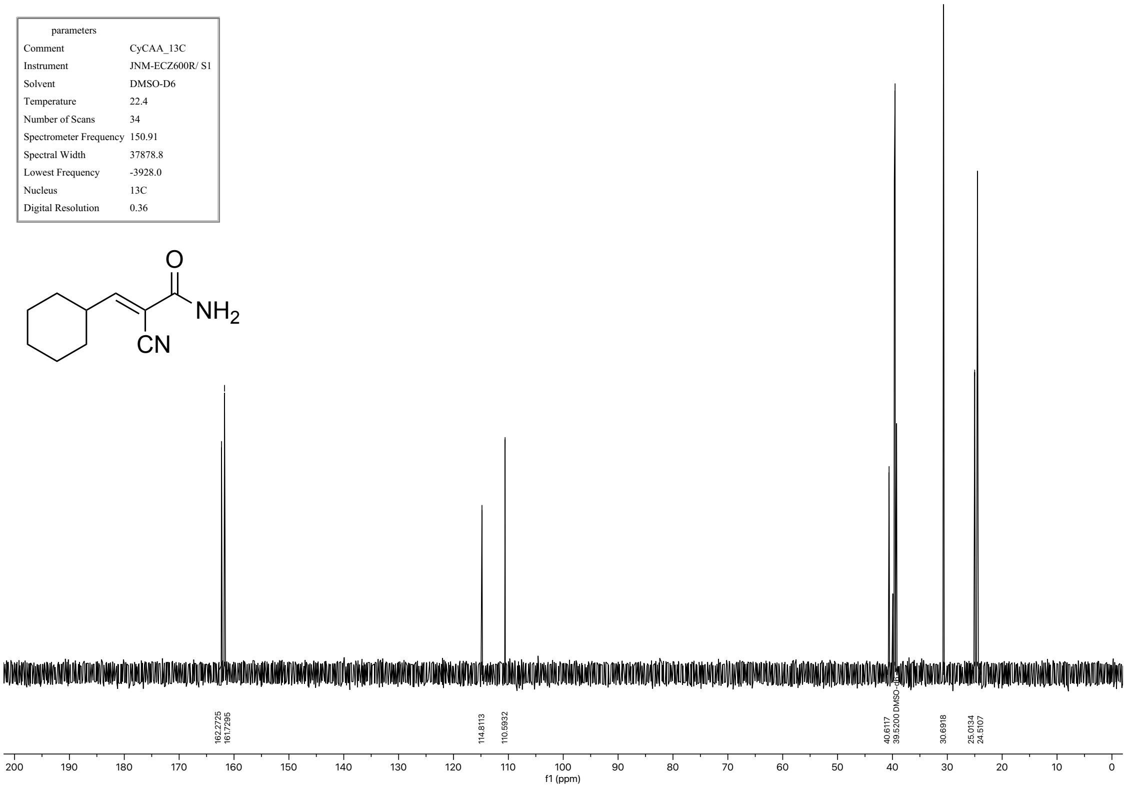

Figure S14. ^{13}C-NMR spectrum of CyCAA.

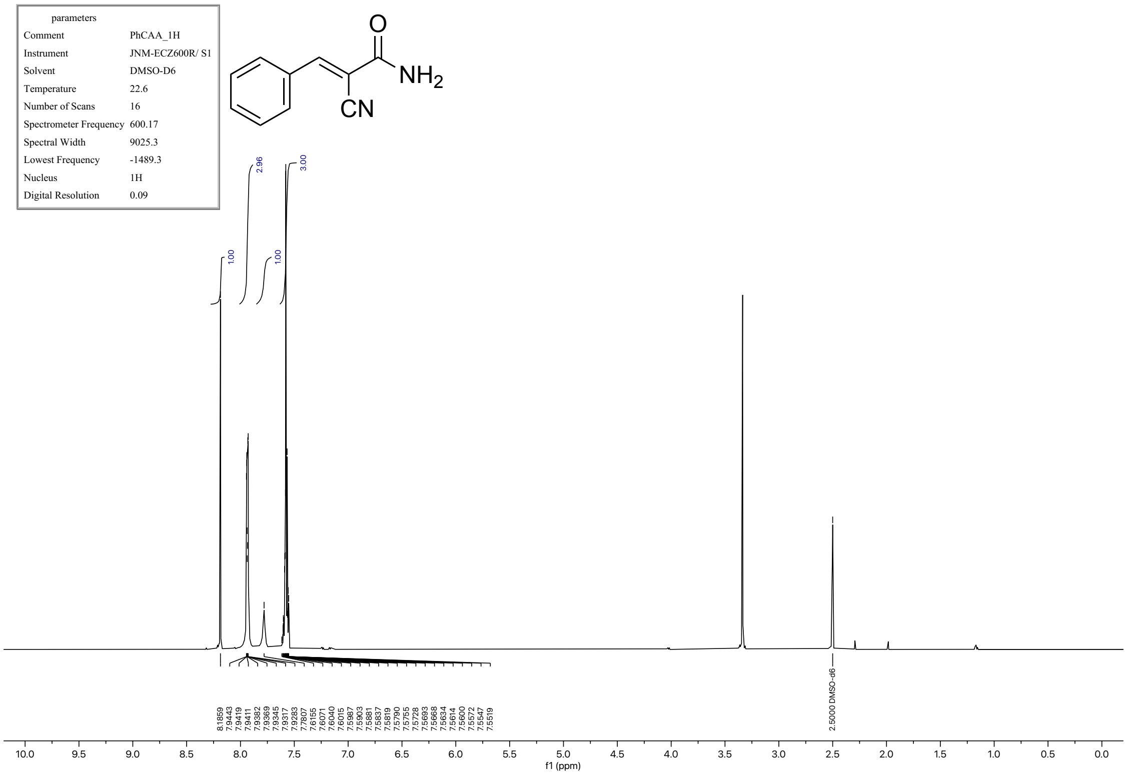

Figure S15. ^{1}H-NMR spectrum of PhCAA.

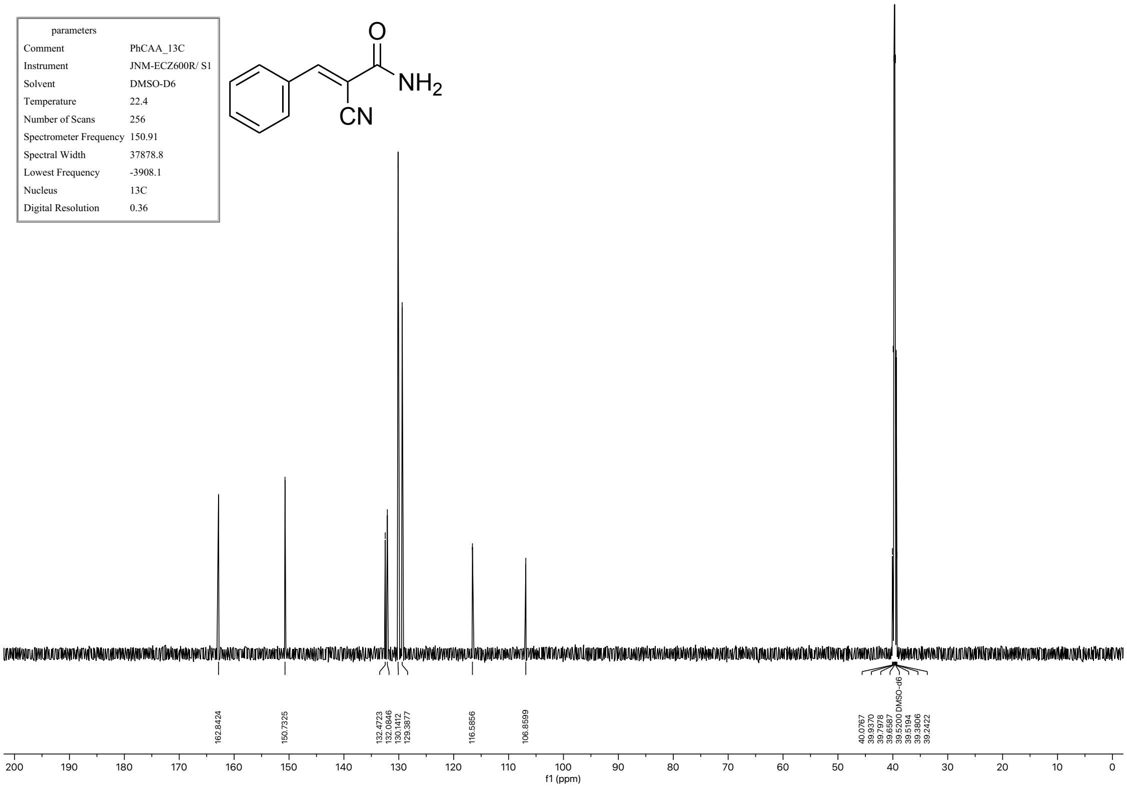

Figure S16. ^{13}C-NMR spectrum of PhCAA.

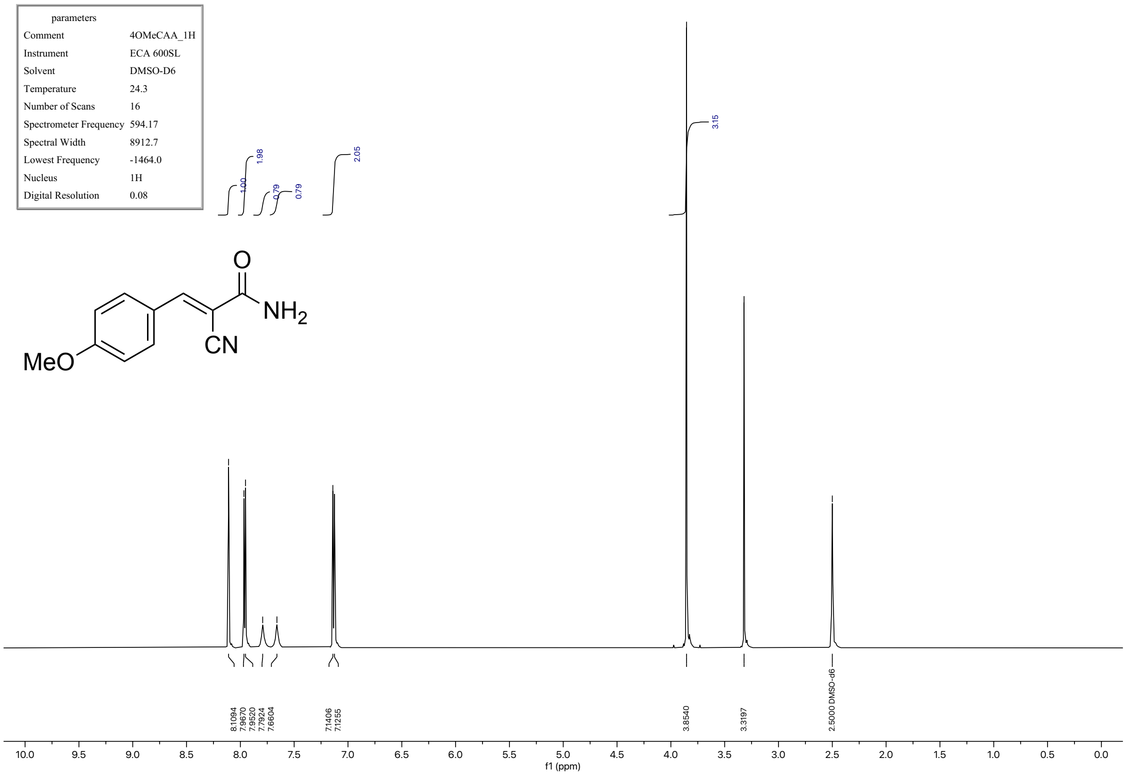

Figure S17. ^{1}H-NMR spectrum of 4OMeCAA.

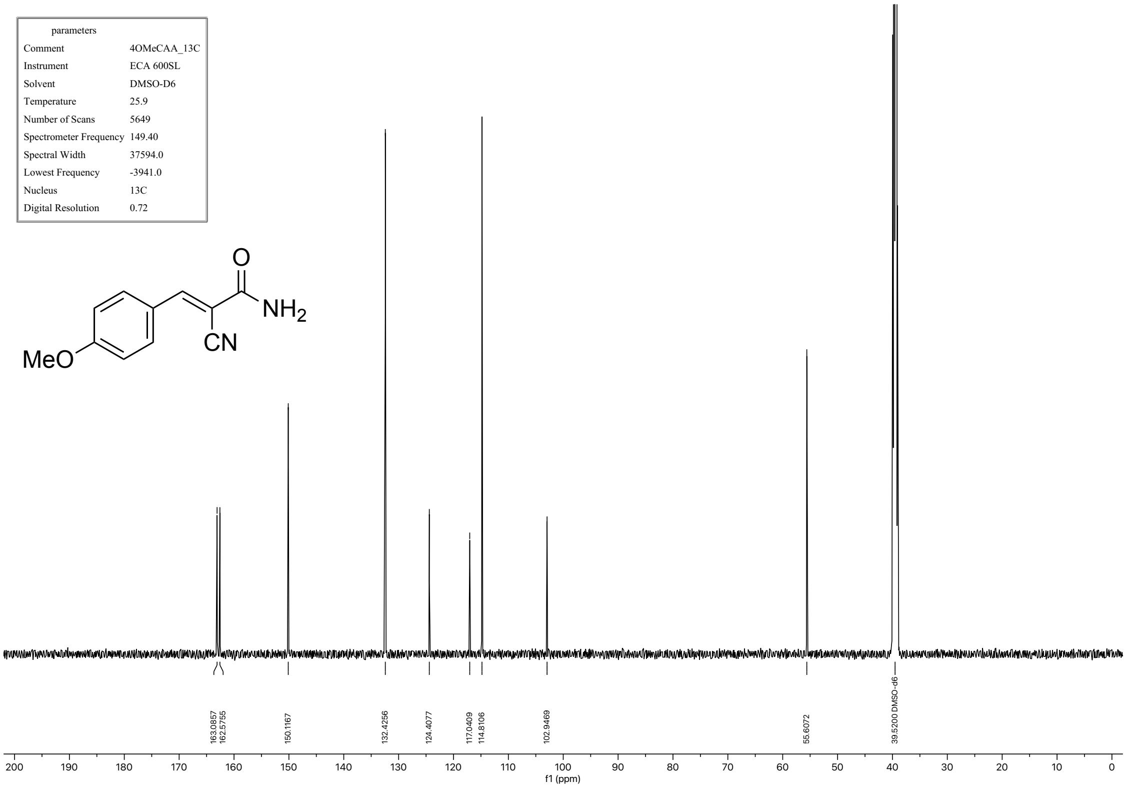

Figure S18. ^{13}C-NMR spectrum of 4OMeCAA.

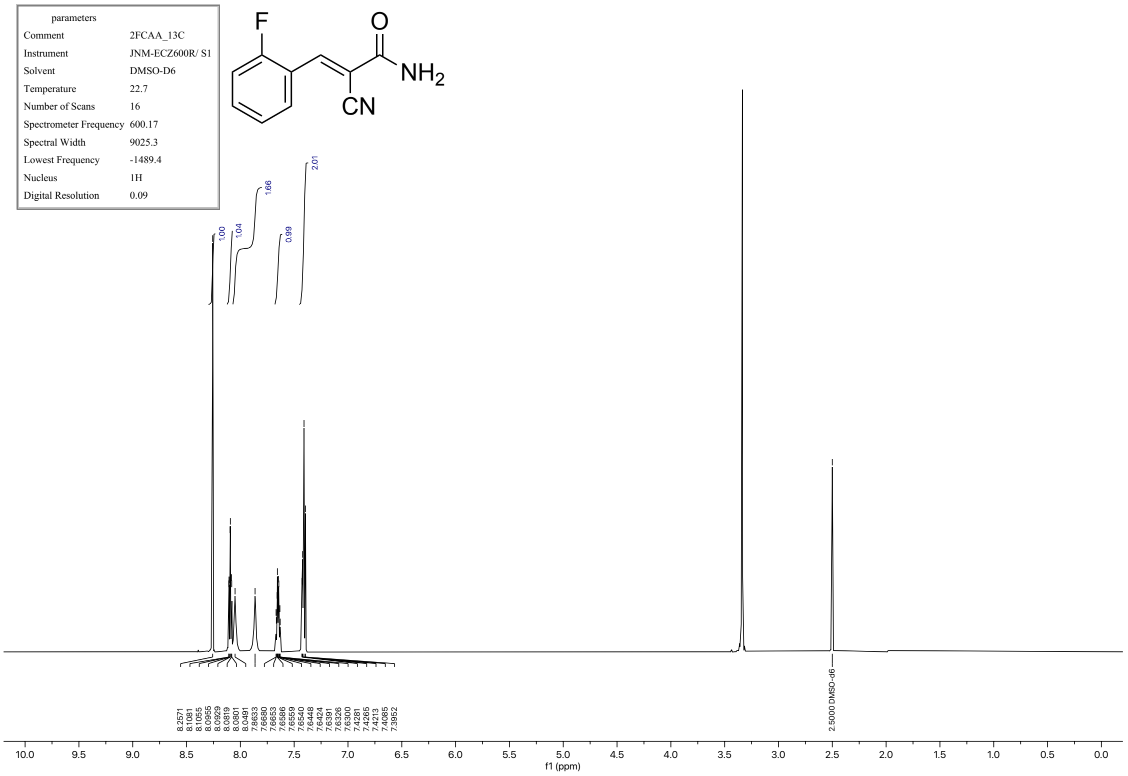

Figure S19. ^{1}H-NMR spectrum of 2FCAA.

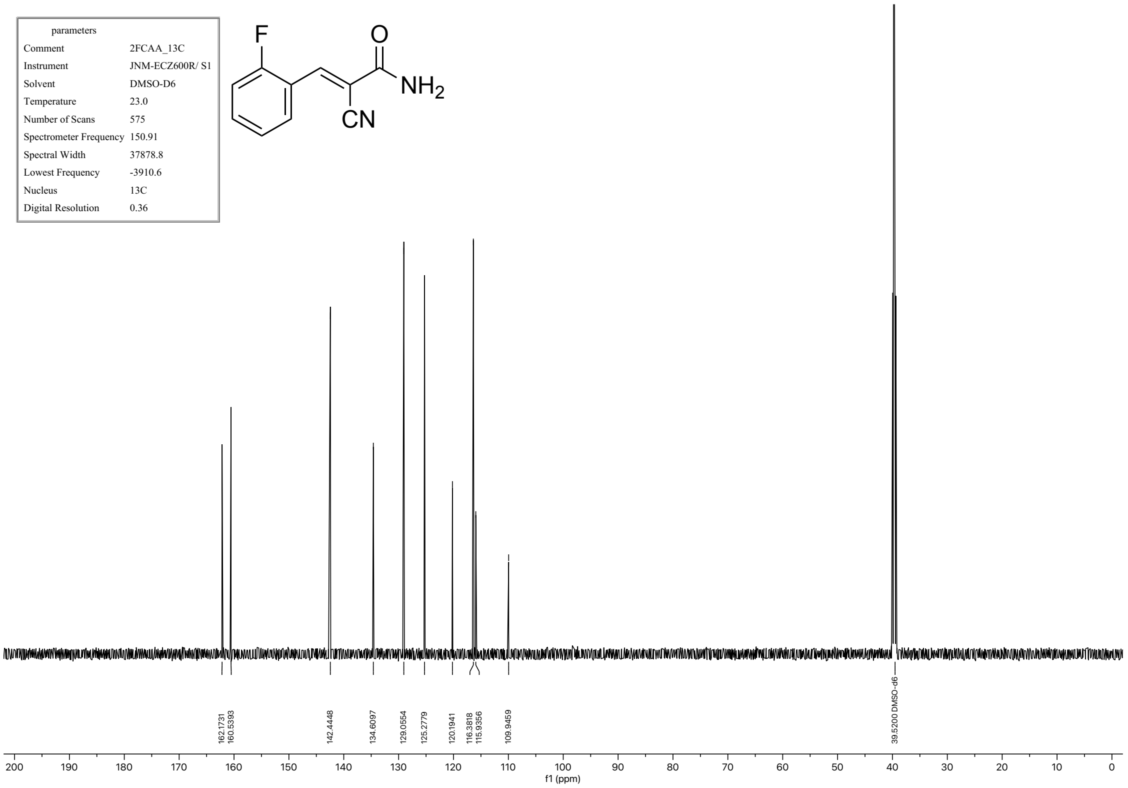

Figure S20. ^{13}C-NMR spectrum of 2FCAA.

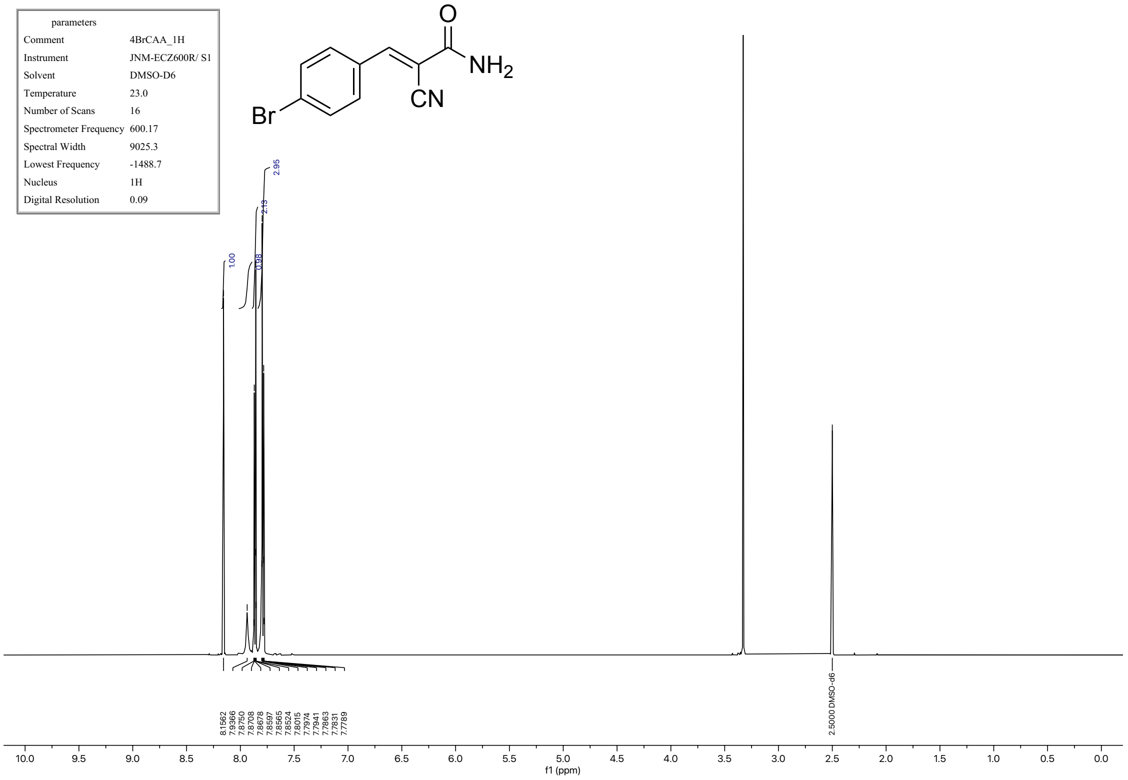

Figure S21. ^{1}H-NMR spectrum of 4BrCAA.

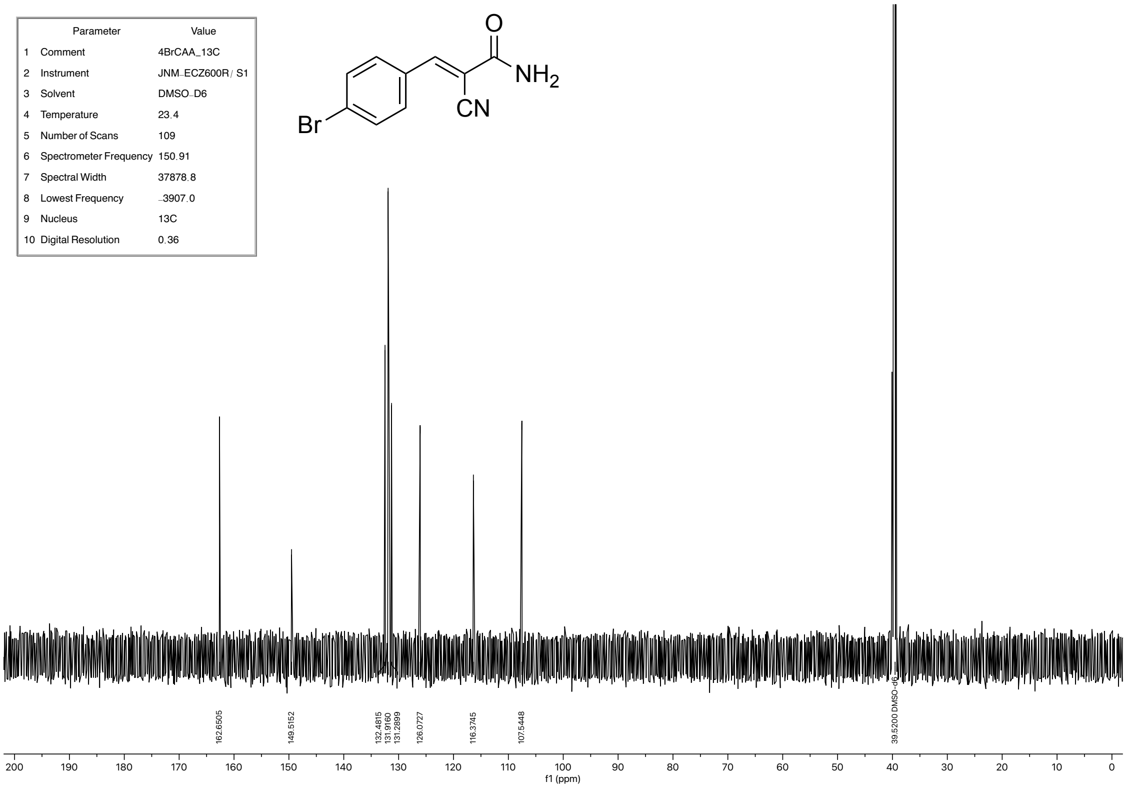

Figure S22. ^{13}C-NMR spectrum of 4BrCAA.

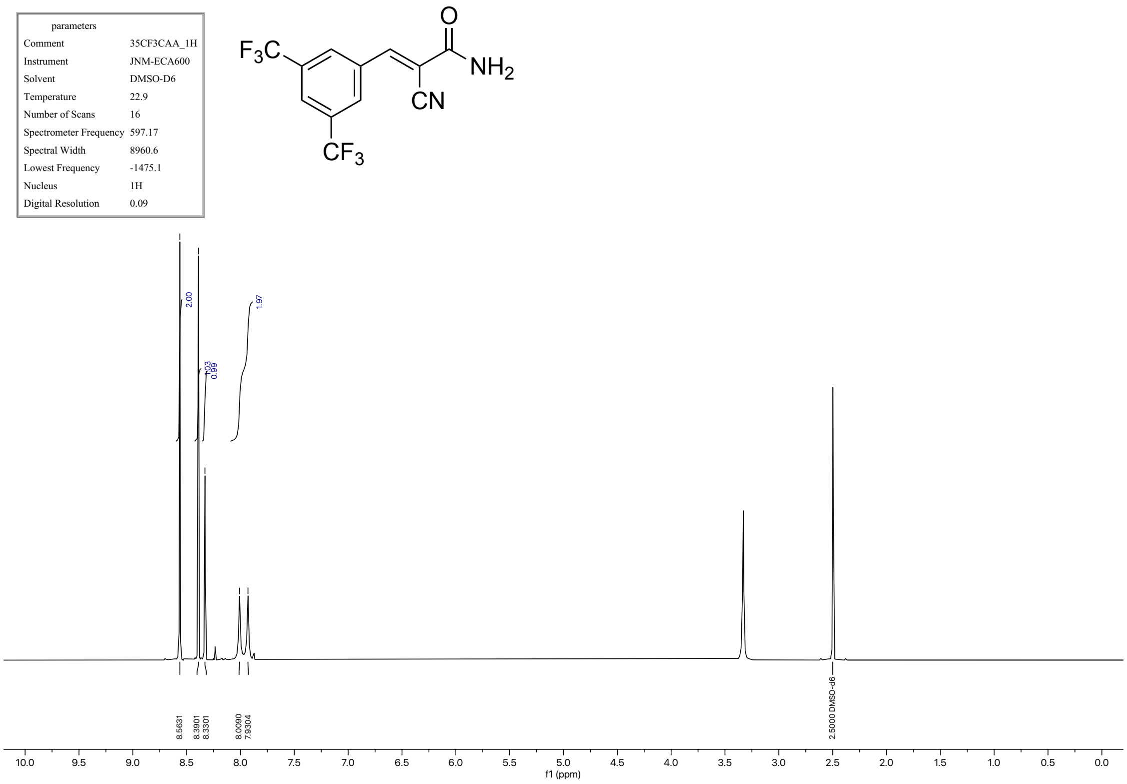

Figure S23. ^{1}H-NMR spectrum of 35CF$_3$CAA.

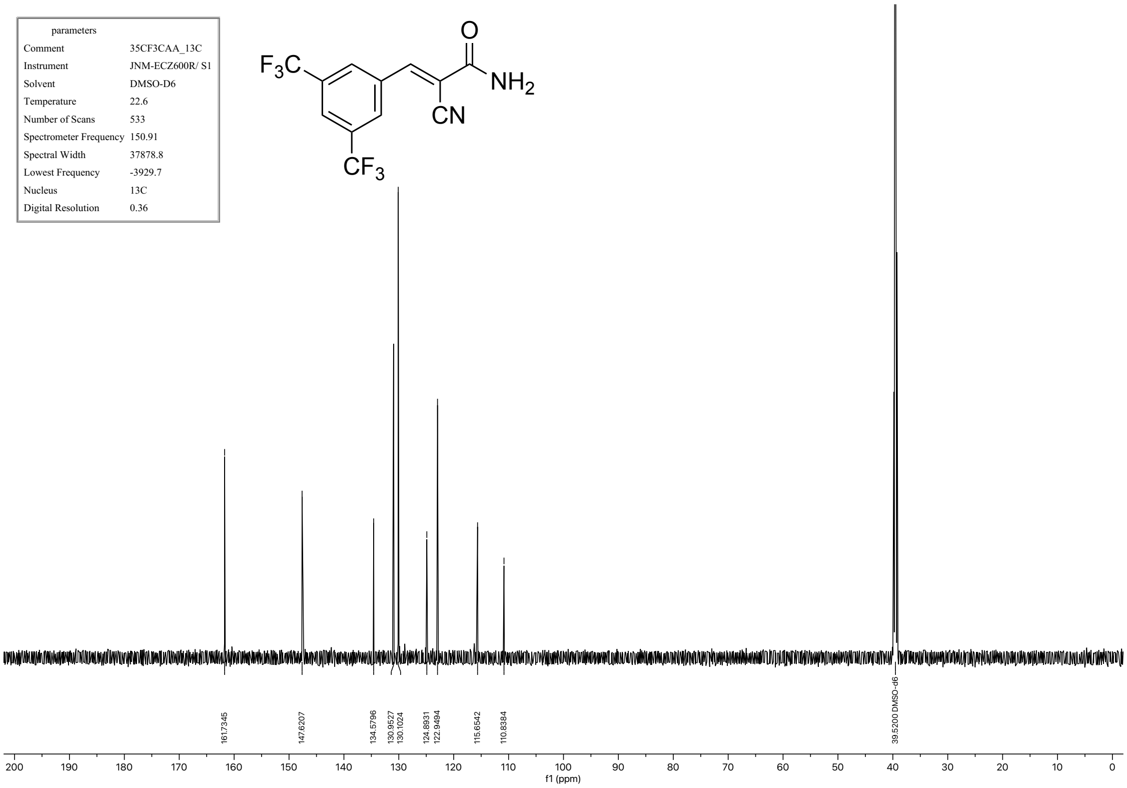

Figure S24. ^{13}C-NMR spectrum of 35CF$_3$CAA.

6. Additional References

(1) Yamakoshi, H.; Dodo, K.; Palonpon, A.; Ando, J.; Fujita, K.; Kawata, S.; Sodeoka, M. Alkyne-Tag Raman Imaging for Visualization of Mobile Small Molecules in Live Cells. *J. Am. Chem. Soc.* **2012**, *134* (51), 20681–20689.

(2) Armesto, D.; Albert, A.; Cano, F. H.; Martín, N.; Ramos, A.; Rodriguez, M.; Segura, J. L.; Seoane, C. A Study on the Scope of the Photochemical Ring Contraction of Substituted 2-Amino-3-Cyano-4H-Pyrans to Cyclobutenes: Crystal Structure of 3-Carbamoyl-3-Cyano-1-Ethoxycarbonyl-4-Isopropyl-2-Phenylcyclobutene. *J. Chem. Soc., Perkin Trans. 1* **1997**, No. 22, 3401–3406.

(3) Zuo, W.; Wang, G.; Yang, S.; Dongxiao; Liu, L.; Li, H.; You, S.; Jia, X. A Chemical-biological Relay Catalytic Method for the Synthesis of (*E*)-2-cyanoacrylamides Based on the Catalysis of Amorphous Porphyrin-MOFs and Nitrile Hydratase. *Adv. Synth. Catal.* **2023**, *365* (18), 3107–3111.

(4) Backus, K. M.; Correia, B. E.; Lum, K. M.; Forli, S.; Horning, B. D.; González-Páez, G. E.; Chatterjee, S.; Lanning, B. R.; Teijaro, J. R.; Olson, A. J.; Wolan, D. W.; Cravatt, B. F. Proteome-Wide Covalent Ligand Discovery in Native Biological Systems. *Nature* **2016**, *534* (7608), 570–574.
